# Supplementary material for: ISLET: individual-specific reference panel recovery improves cell-type-specific inference
Source: Genome Biol. 2023 Jul 26;24:174. doi: 10.1186/s13059-023-03014-8 (PMC10373385; doi:10.1186/s13059-023-03014-8)
Supplement: Supplementary file 1 — Additional file 1. Simulation: reference panel estimation. [file 13059_2023_3014_MOESM1_ESM.pdf]

ISLET: individual-specific reference panel recovery improves cell-type-specific inference

Additional File 1

Simulation: reference panel estimation

Hao Feng*, Guanqun Meng, Tong Lin, Hemang Parikh,
Yue Pan, Ziyi Li, Jeffrey Krischer and Qian Li*

Contents

1 Simulation Results:
solve for individual-specific reference panels **2**
1.1 Estimated Reference Panels 2
1.2 Reference estimation: using true versus estimated proportions. 3
1.3 Stratified NMSE 4

1 Simulation Results: solve for individual-specific reference panels

1.1 Estimated Reference Panels

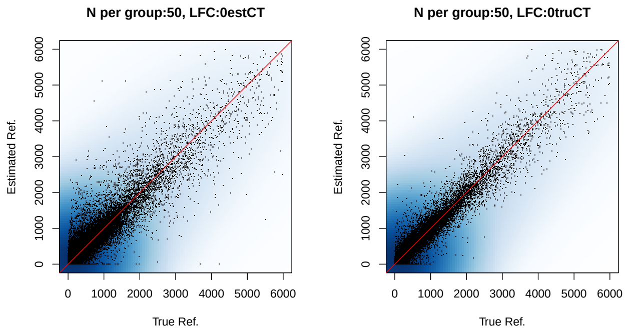

(a) 50 samples per group and no DEG.

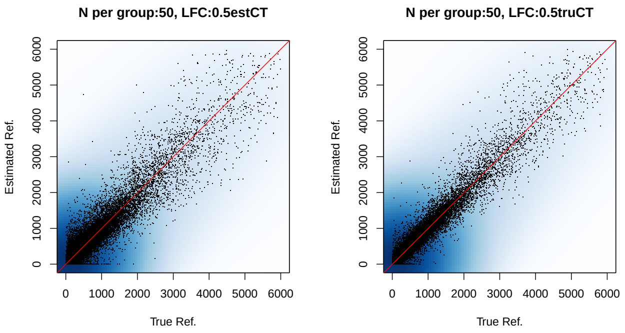

(b) 50 samples per group, LFC equals 0.5

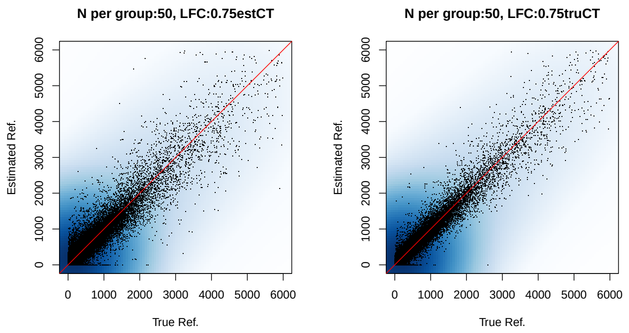

(c) 50 samples per group, LFC equals 0.75

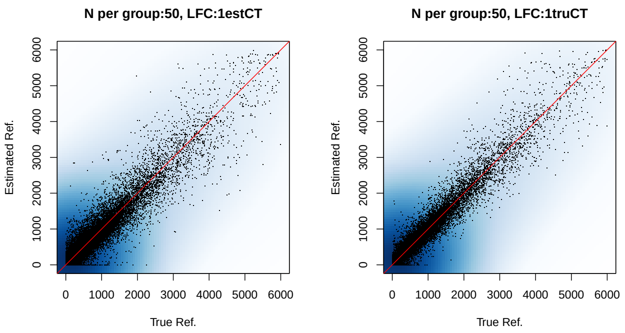

(d) 50 samples per group, LFC equals 1

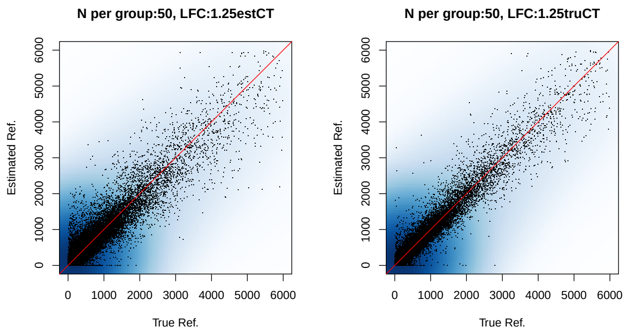

(e) 50 samples per group, LFC equals 1.25

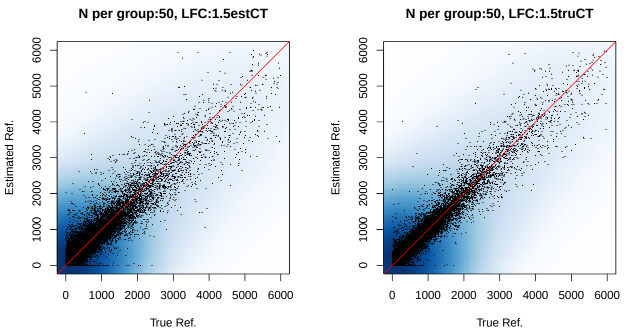

(f) 50 samples per group, LFC equals 1.5

Figure S1: Estimated individual-specific and cell-specific reference panels from ISLET versus truth, under various LFC from (**a**) to (**f**). For each panel, the plot on the left shows the result of using the estimated cell type proportions as ISLET inputs, while plot on right use the true cell type proportions as ISLET input.

1.2 Reference estimation: using true versus estimated proportions.

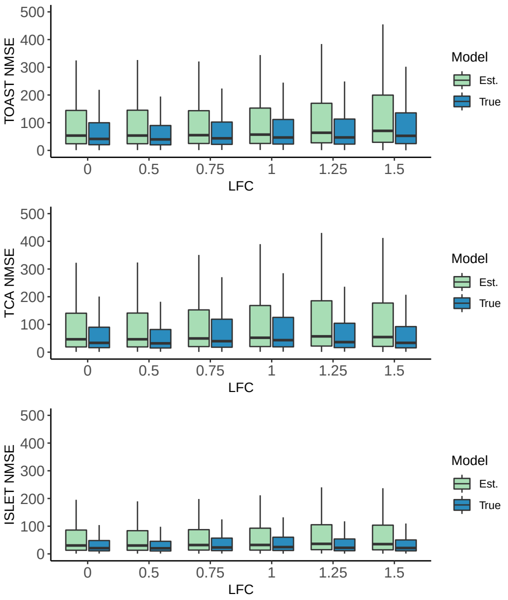

(a) 25 samples per group

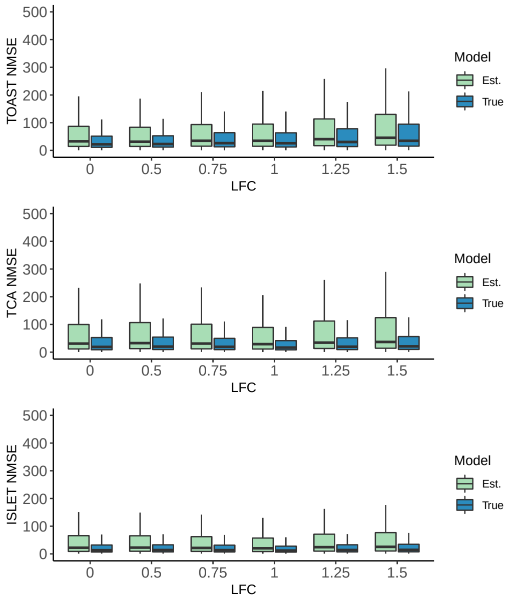

(b) 50 samples per group

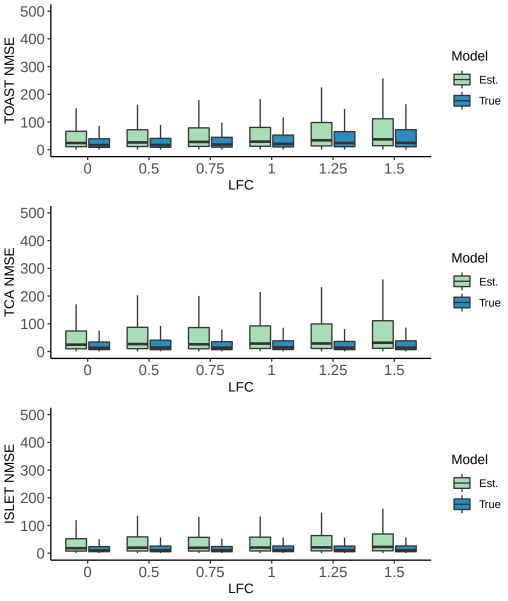

(c) 75 samples per group

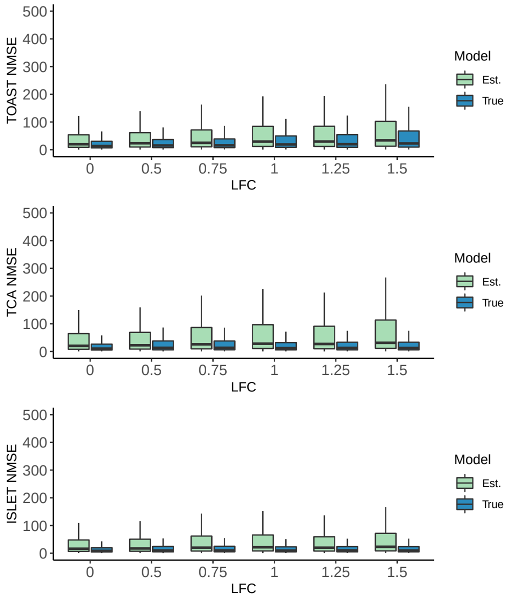

(d) 100 samples per group

Figure S2: Normalized Mean Square Error (NMSE) comparison when using the true versus estimated cell type proportions, as input, for TOAST, TCA, and ISLET. Simulations were conducted at various LFCs and sample sizes. Comparisons of NMSE are side-by-side in each scenario: the left bar represents NMSE using the estimated cell type proportions, and the right bar represents NMSE using the true cell type proportions.

1.3 Stratified NMSE

Sample size = 25.

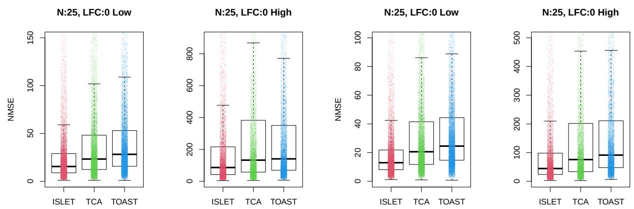

(a) No csDEG (LFC=0).

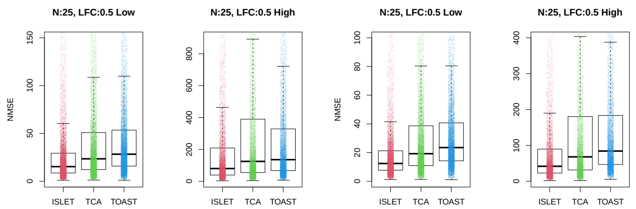

(b) LFC = 0.5

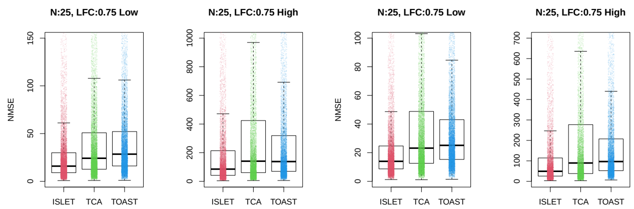

(c) LFC = 0.75.

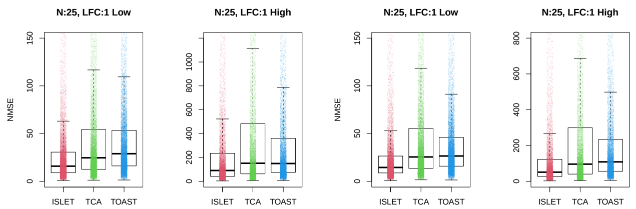

(d) LFC = 1

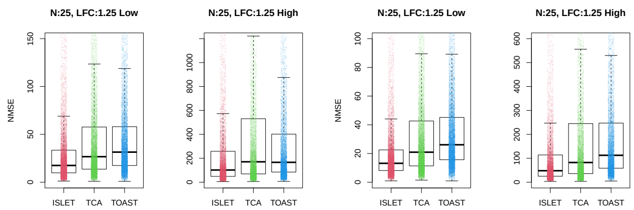

(e) LFC = 1.25

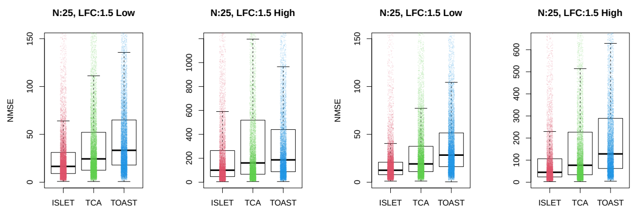

(f) LFC = 1.5

Figure S3: Stratified NMSE comparing TOAST, TCA, and ISLET under different LFC from (**a**) to (**f**). The stratification (low and high) is based on RNA-seq gene expressions count value. For each panel, the two plots on left show the results of using the estimated cell type proportions as inputs, and two plots on right show the results of using the estimated cell type proportions as inputs. Sample size = 25.

Sample size = 50

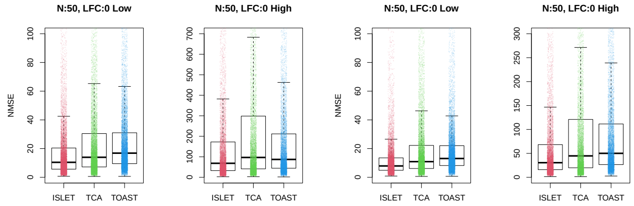

(a) No csDEG (LFC=0).

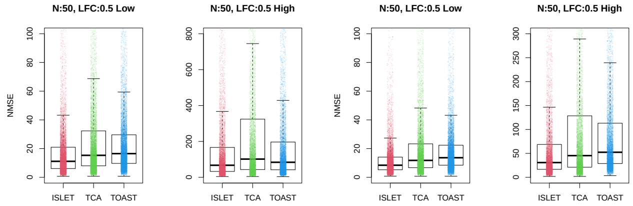

(b) LFC = 0.5

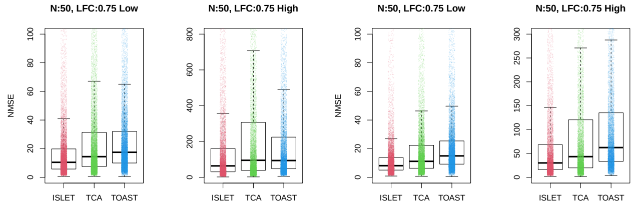

(c) LFC = 0.75.

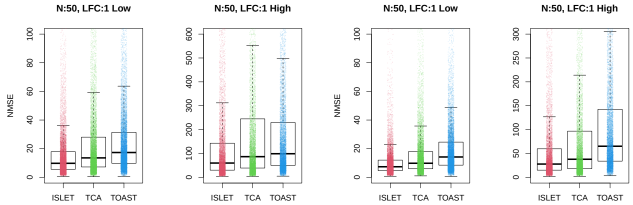

(d) LFC = 1

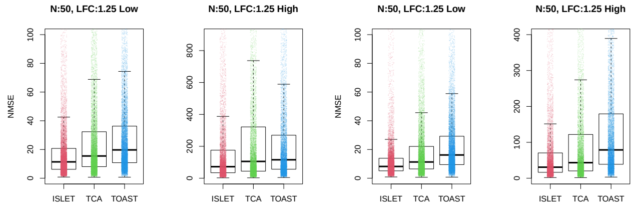

(e) LFC = 1.25

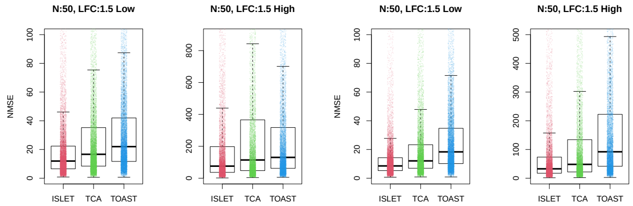

(f) LFC = 1.5

Figure S4: Stratified NMSE comparing TOAST, TCA, and ISLET under different LFC from (**a**) to (**f**). The stratification (low and high) is based on RNA-seq gene expressions count value. For each panel, the two plots on left show the results of using the estimated cell type proportions as inputs, and two plots on right show the results of using the estimated cell type proportions as inputs. Sample size = 50.

Sample size = 75

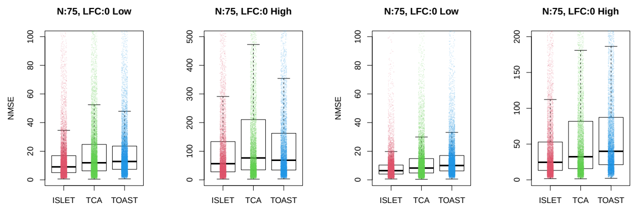

(a) No csDEG (LFC=0).

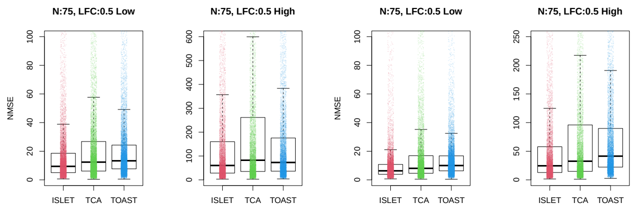

(b) LFC = 0.5

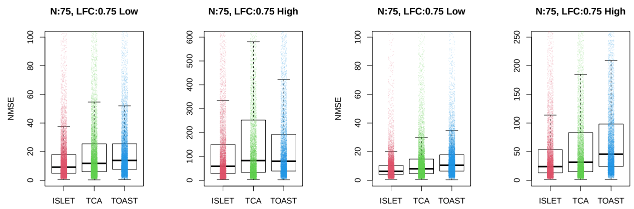

(c) LFC = 0.75.

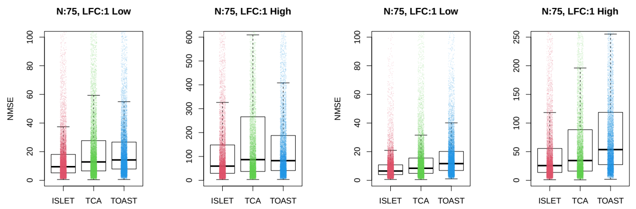

(d) LFC = 1

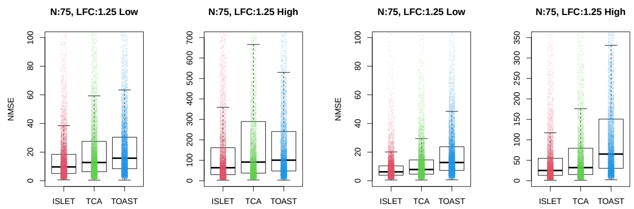

(e) LFC = 1.25

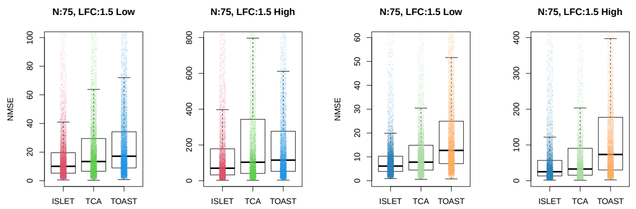

(f) LFC = 1.5

Figure S5: Stratified NMSE comparing TOAST, TCA, and ISLET under different LFC from (**a**) to (**f**). The stratification (low and high) is based on RNA-seq gene expressions count value. For each panel, the two plots on left show the results of using the estimated cell type proportions as inputs, and two plots on right show the results of using the estimated cell type proportions as inputs. Sample size = 75.

Sample size = 100

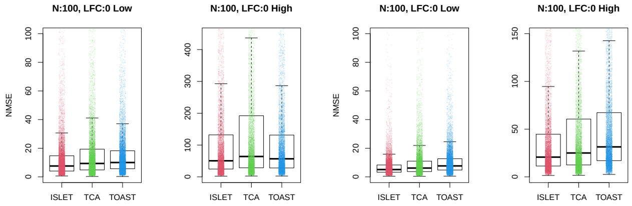

(a) No csDEG (LFC=0).

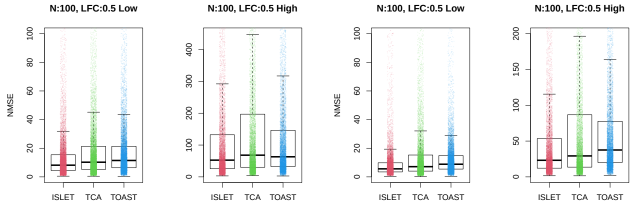

(b) LFC = 0.5

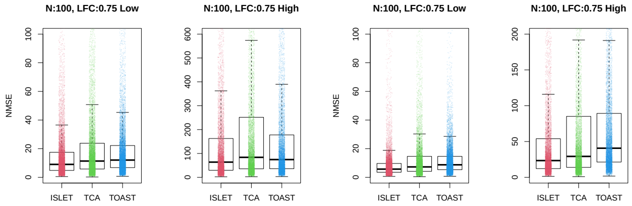

(c) LFC = 0.75.

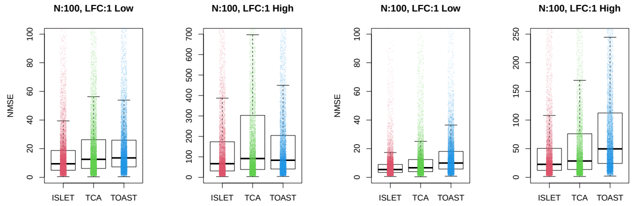

(d) LFC = 1

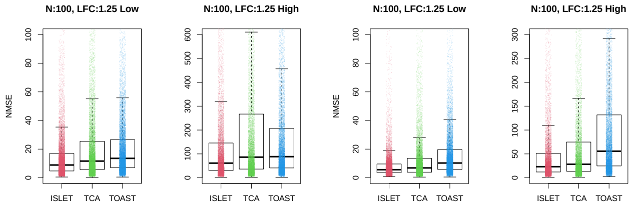

(e) LFC = 1.25

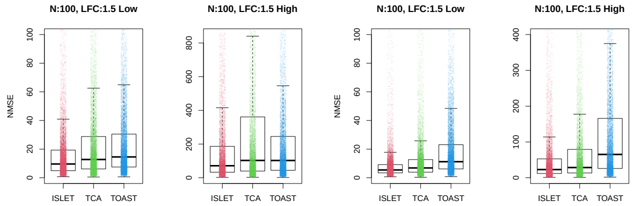

(f) LFC = 1.5

Figure S6: Stratified NMSE comparing TOAST, TCA, and ISLET under different LFC from (**a**) to (**f**). The stratification (low and high) is based on RNA-seq gene expressions count value. For each panel, the two plots on left show the results of using the estimated cell type proportions as inputs, and two plots on right show the results of using the estimated cell type proportions as inputs. Sample size = 100.

The figure below shows the normalized MSE for solving refernce panel, in an individual-wise fashion, for TOAST using 'nnls' function. For each individual reference panel estimation, only the replicate samples for that specific individual are utilized in estimation. Results indicate unstable and worse estimation for TOAST. And ISLET maintains the lowest NMSE.

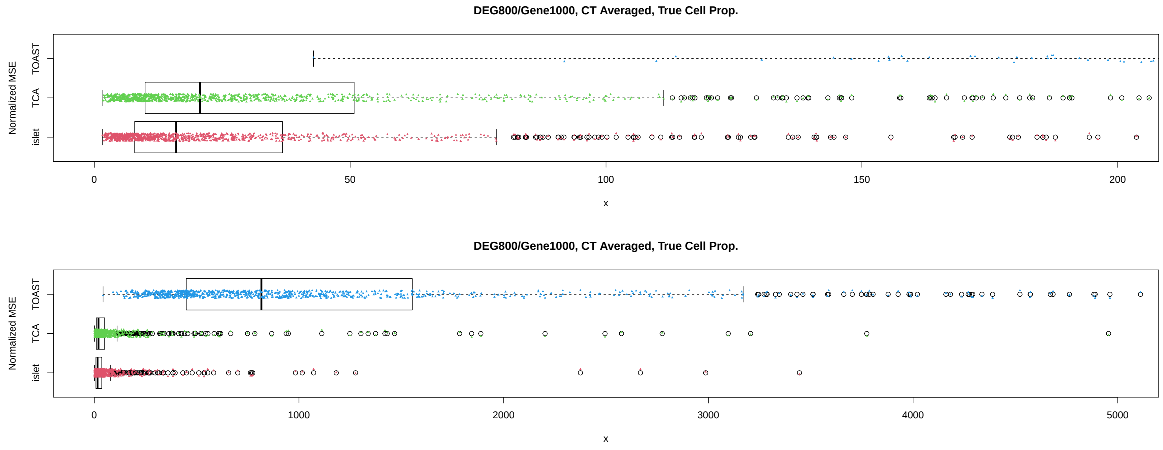

Figure S7: NMSE comparing TOAST, TCA, and ISLET. Here, TOAST is utilized in an individual-wise fashion. Top panel: zoomed-in NMSE. Bottom panel: overall NMSE. The zoomed-in (top panel) shows the area toward the far left end of the overall NMSE (bottom panel).
