# Supplementary material for: ISLET: individual-specific reference panel recovery improves cell-type-specific inference
Source: Genome Biol. 2023 Jul 26;24:174. doi: 10.1186/s13059-023-03014-8 (PMC10373385; doi:10.1186/s13059-023-03014-8)
Supplement: Supplementary file 2 — Additional file 2. Simulation: csDEG test in mean. [file 13059_2023_3014_MOESM2_ESM.pdf]

ISLET: individual-specific reference panel recovery improves cell-type-specific inference

Additional File 2

Simulation: csDEG test in mean

Hao Feng*, Guanqun Meng, Tong Lin, Hemang Parikh,
Yue Pan, Ziyi Li, Jeffrey Krischer and Qian Li*

Contents

**1 Simulation Results:
cell-type-specific Differentially Expressed Genes (csDEG) Identification 2**

1.1 TDR (cell-type-specific) 2
1.2 ROC (cell-type-specific) 6
1.3 Sensitivity versus FDR 10
1.4 Sensitivity (cell-type-specific) 14
1.5 Power comparison using true and estimated proportions. 15
1.6 Precision, ROC, FDR and power comparison using true and estimated proportions. . . . 16
1.7 Comparison under small sample size. 17

1 Simulation Results: cell-type-specific Differentially Expressed Genes (csDEG) Identification

1.1 TDR (cell-type-specific)

The TDR curves are shown, for each cell type, under exhaustive combination of sample size and effect size, for all six methods.

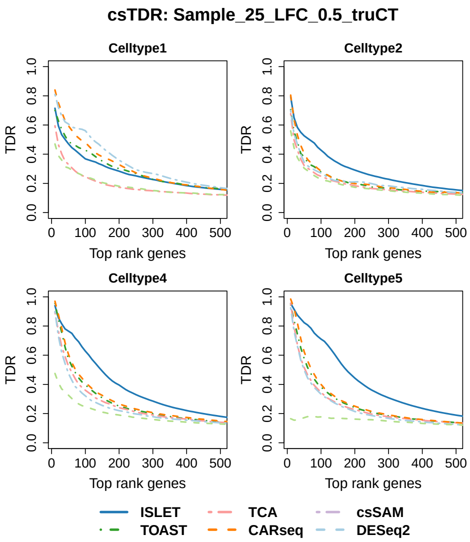

(a) LFC: 0.5

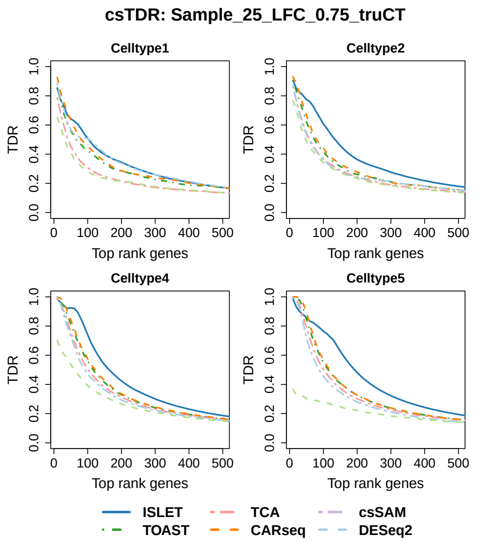

(b) LFC: 0.75

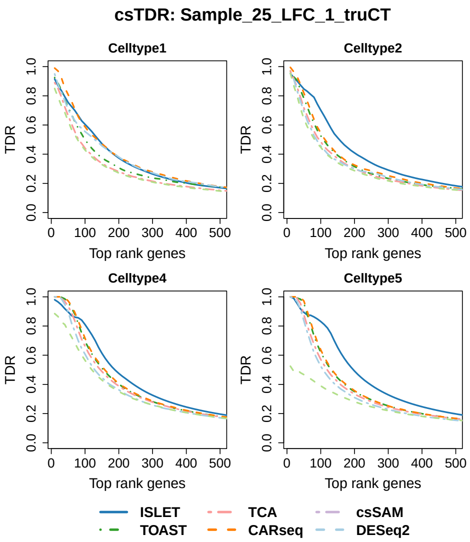

(c) LFC: 1

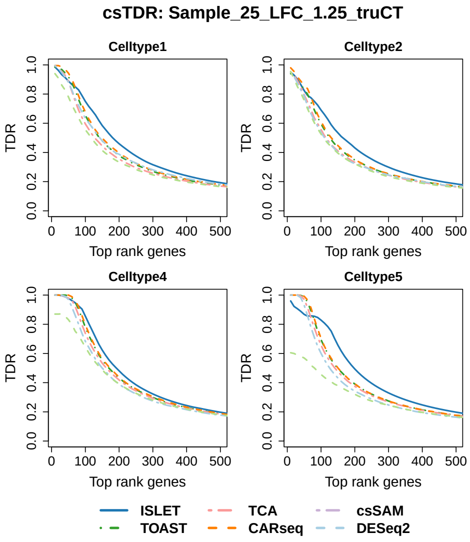

(d) LFC: 1.25

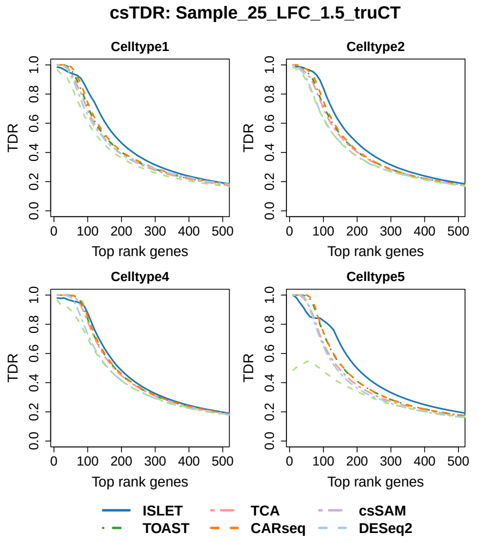

(e) LFC: 1.5

Figure S8: TDR plots for 4 cell types that contain csDEG, for sample size N=25 per group. (a)-(e) represent LFC 0.5 to 1.5.

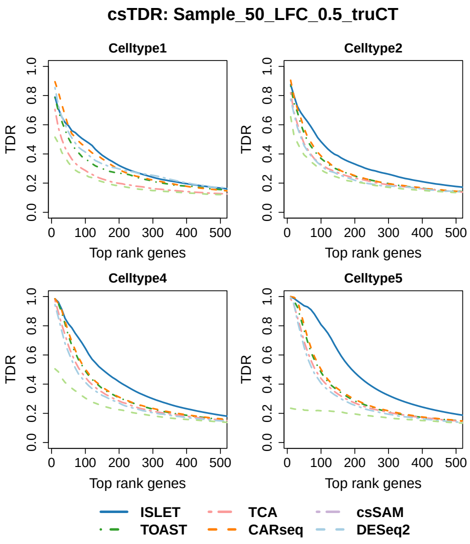

(a) LFC: 0.5

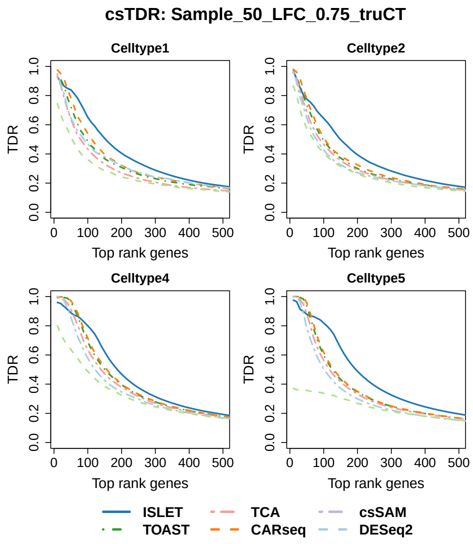

(b) LFC: 0.75

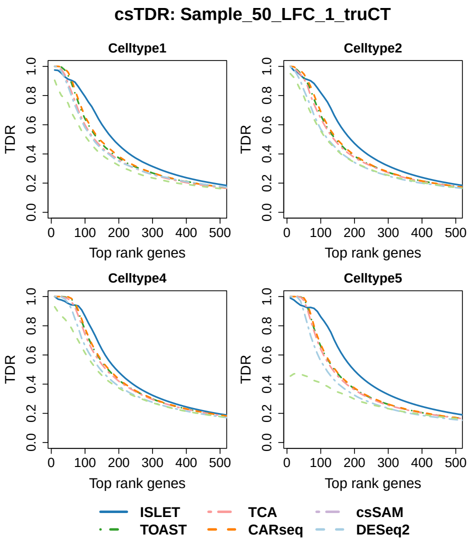

(c) LFC: 1

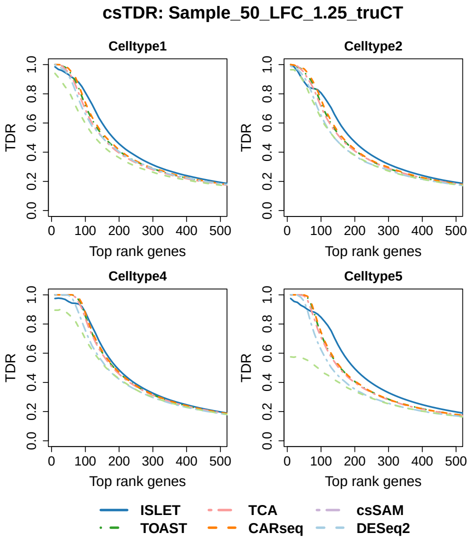

(d) LFC: 1.25

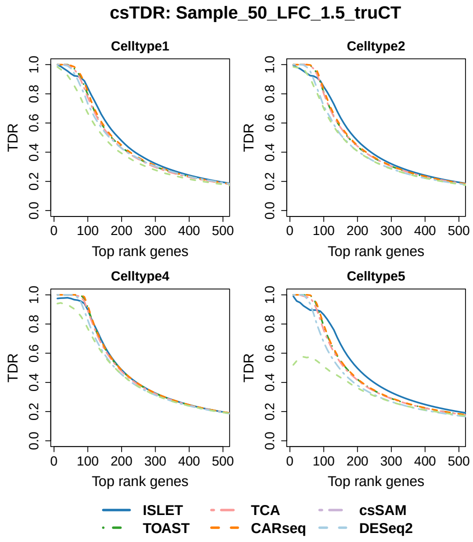

(e) LFC: 1.5

Figure S9: TDR plots for 4 cell types that contain csDEG, for sample size N=50 per group. (a)-(e) represent LFC 0.5 to 1.5.

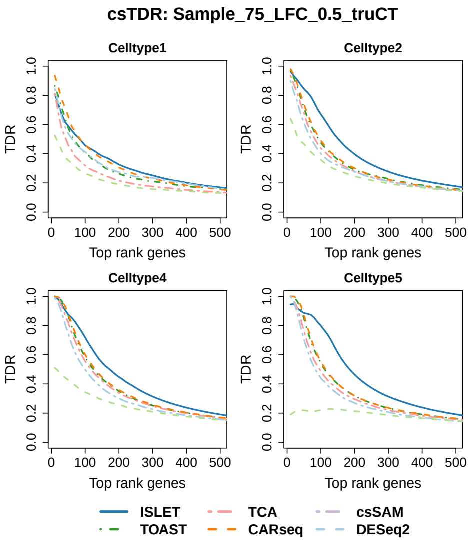

(a) LFC: 0.5

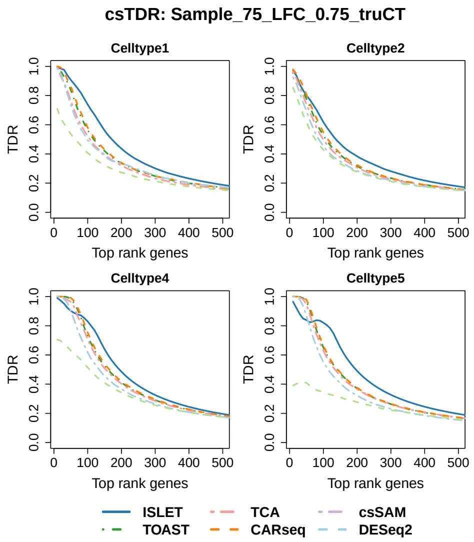

(b) LFC: 0.75

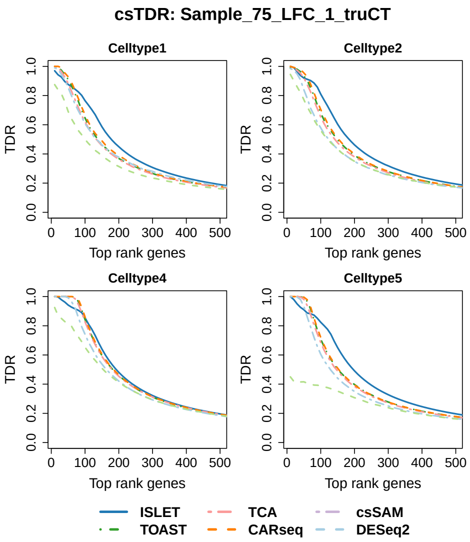

(c) LFC: 1

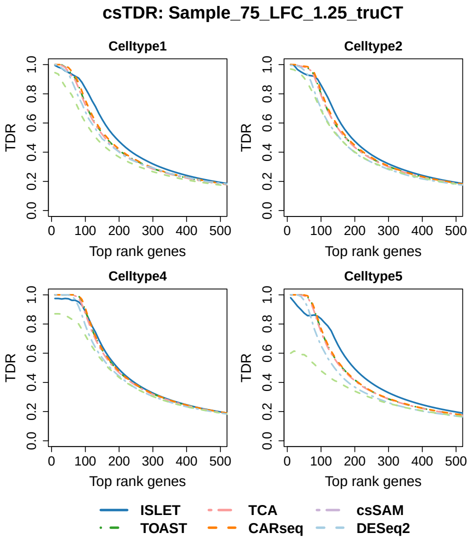

(d) LFC: 1.25

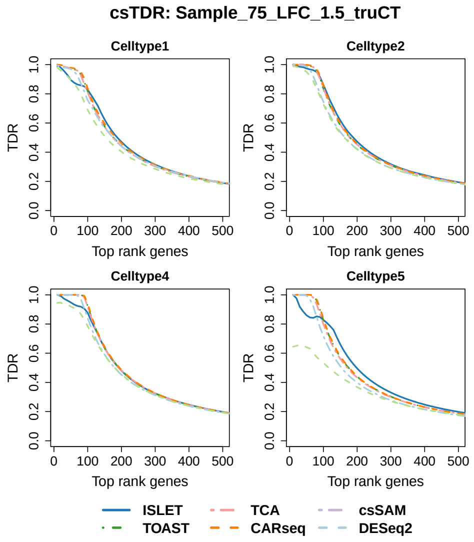

(e) LFC: 1.5

Figure S10: TDR plots for 4 cell types that contain csDEG, for sample size N=75 per group. (a)-(e) represent LFC 0.5 to 1.5.

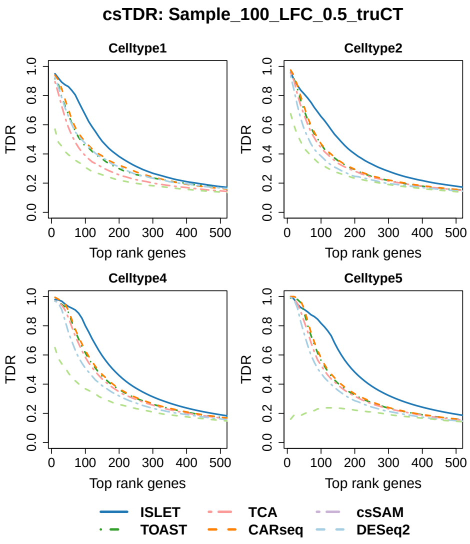

(a) LFC: 0.5

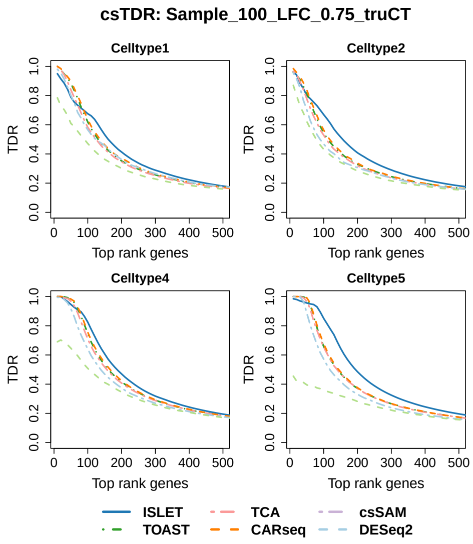

(b) LFC: 0.75

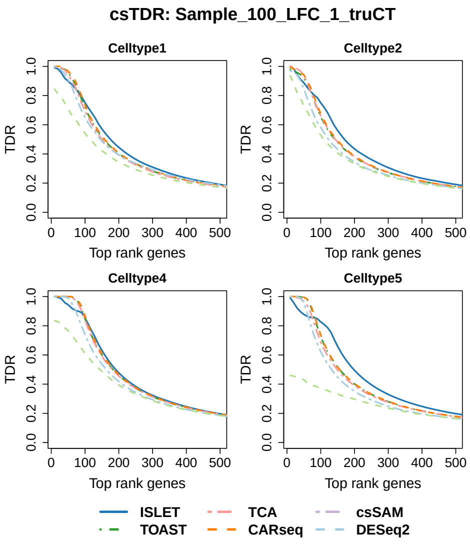

(c) LFC: 1

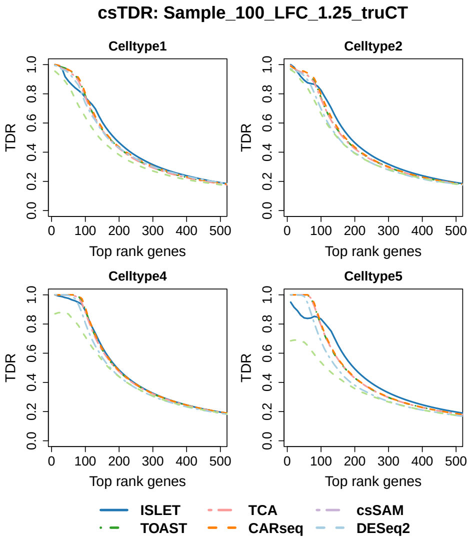

(d) LFC: 1.25

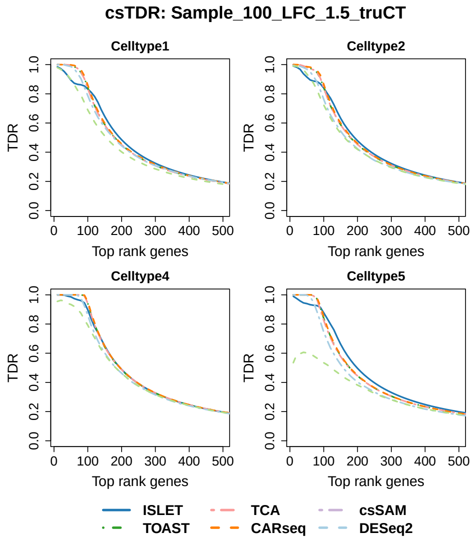

(e) LFC: 1.5

Figure S11: TDR plots for 4 cell types that contain csDEG, for sample size N=100 per group. (a)-(e) represent LFC 0.5 to 1.5.

1.2 ROC (cell-type-specific)

The ROC curves, together with AUC values, are shown under exhaustive combination of sample size and effect size, for all six methods.

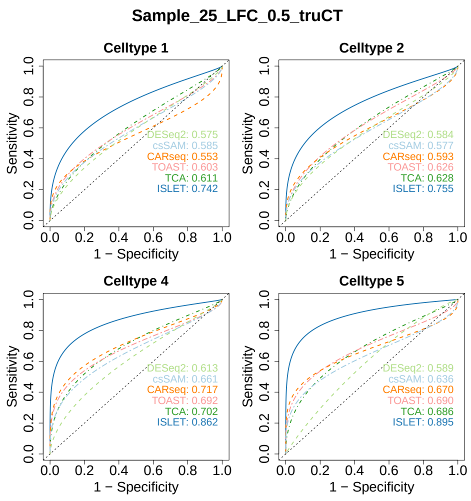

(a) LFC: 0.5

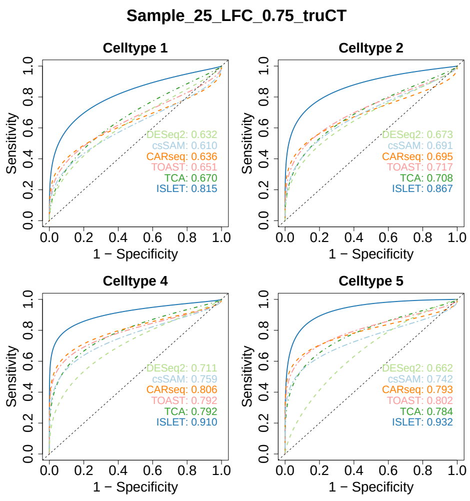

(b) LFC: 0.75

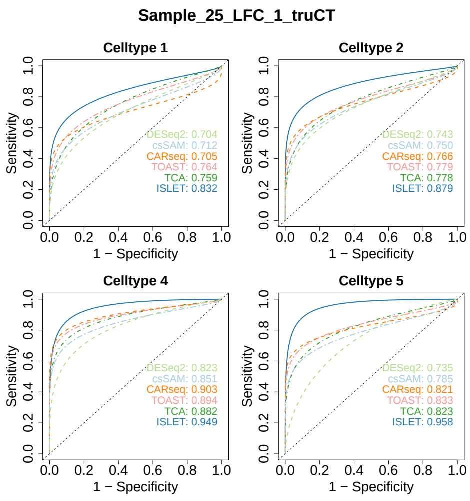

(c) LFC: 1

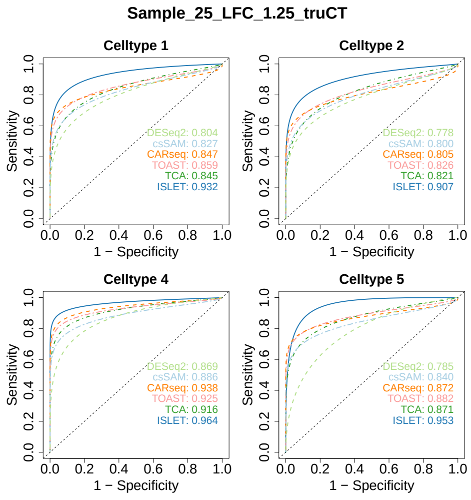

(d) LFC: 1.25

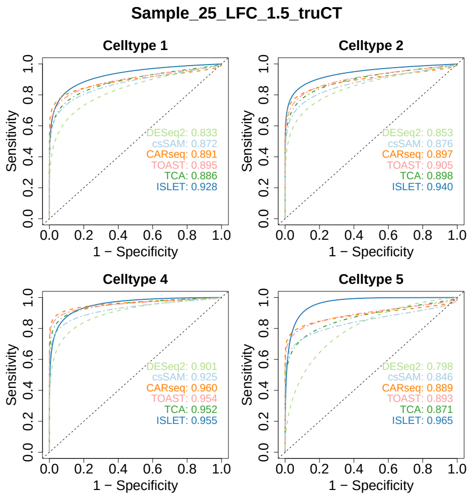

(e) LFC: 1.5

Figure S12: ROC curve plots for 4 cell types that contain csDEG, for sample size N=25 per group. (a)-(e) represent LFC 0.5 to 1.5.

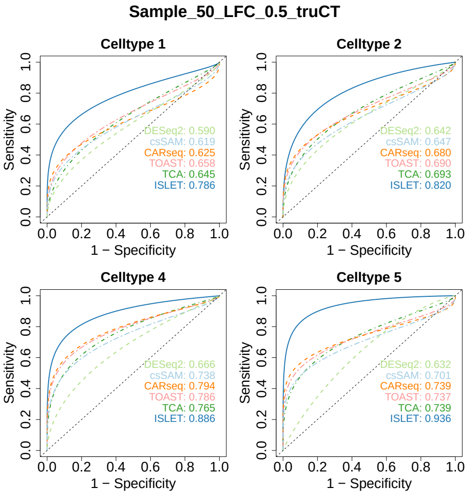

(a) LFC: 0.5

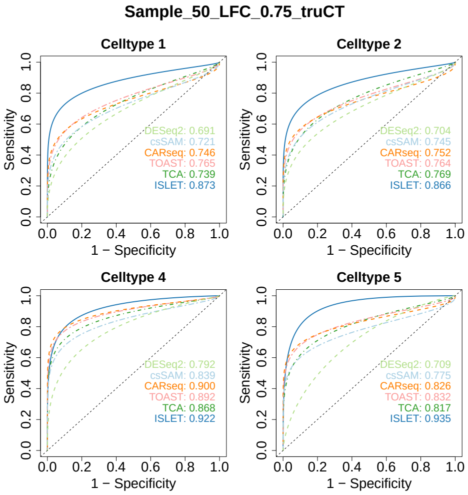

(b) LFC: 0.75

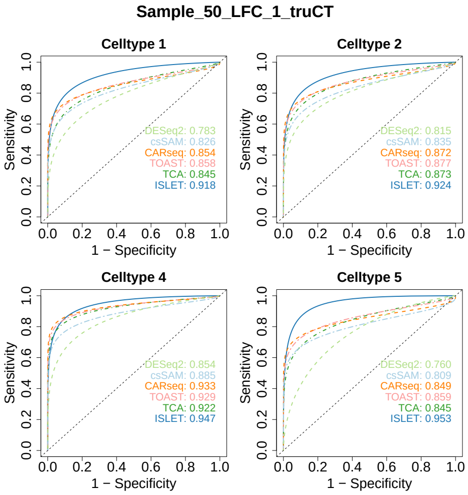

(c) LFC: 1

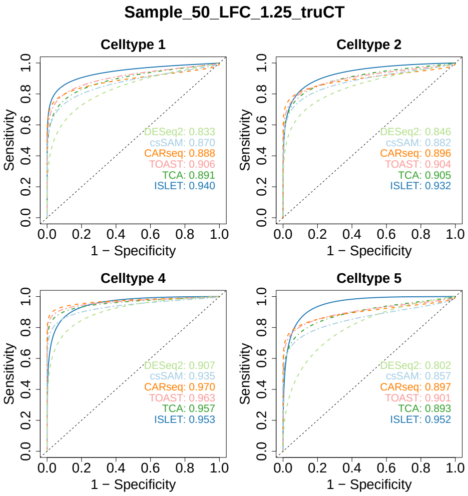

(d) LFC: 1.25

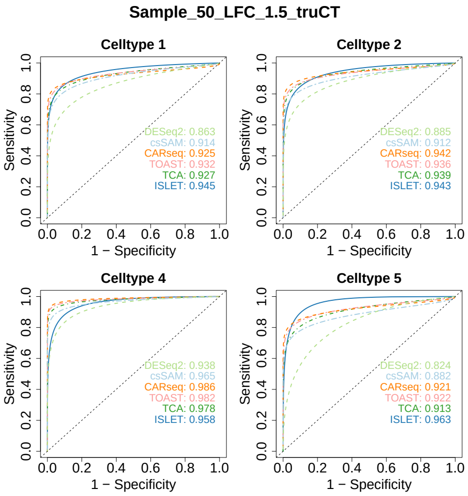

(e) LFC: 1.5

Figure S13: ROC curve plots for 4 cell types that contain csDEG, for sample size N=50 per group. (a)-(e) represent LFC 0.5 to 1.5.

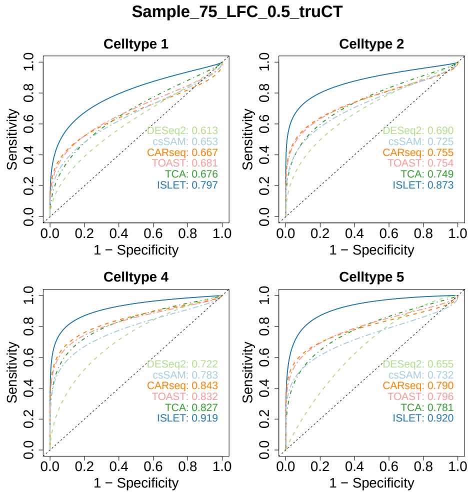

(a) LFC: 0.5

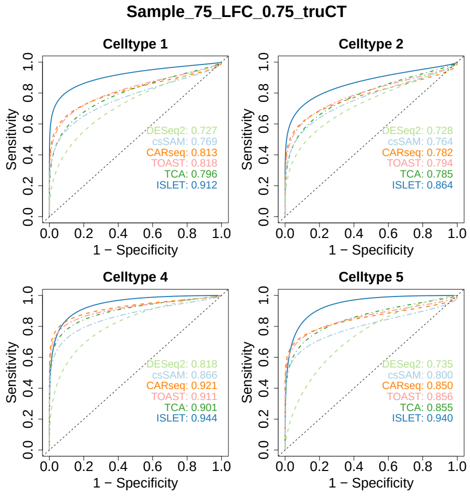

(b) LFC: 0.75

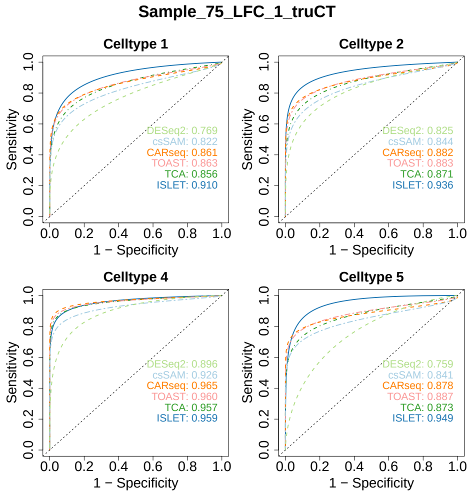

(c) LFC: 1

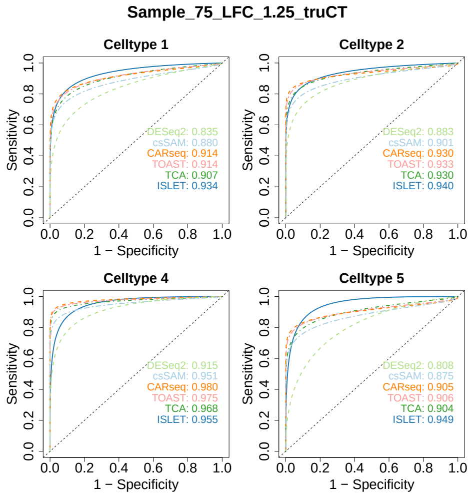

(d) LFC: 1.25

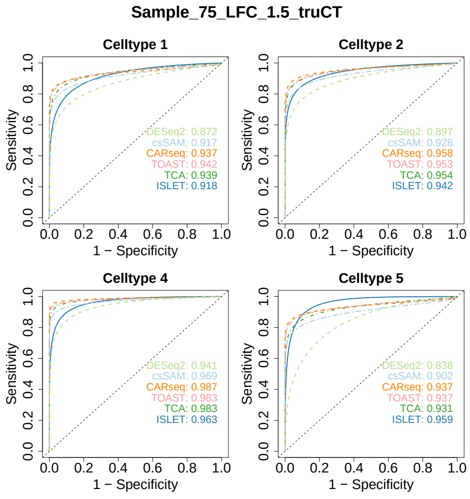

(e) LFC: 1.5

Figure S14: ROC curve plots for 4 cell types that contain csDEG, for sample size N=75 per group. (a)-(e) represent LFC 0.5 to 1.5.

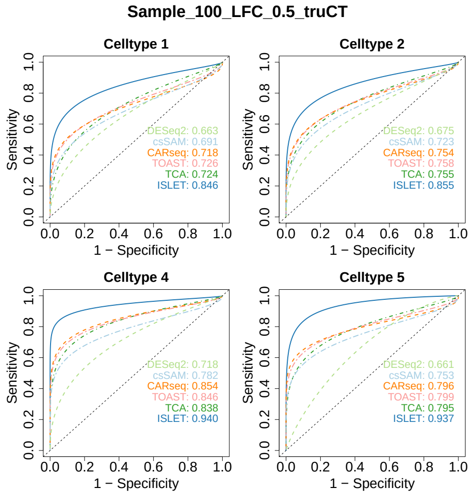

(a) LFC: 0.5

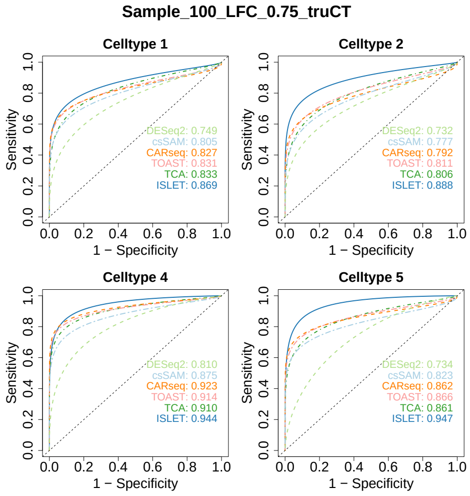

(b) LFC: 0.75

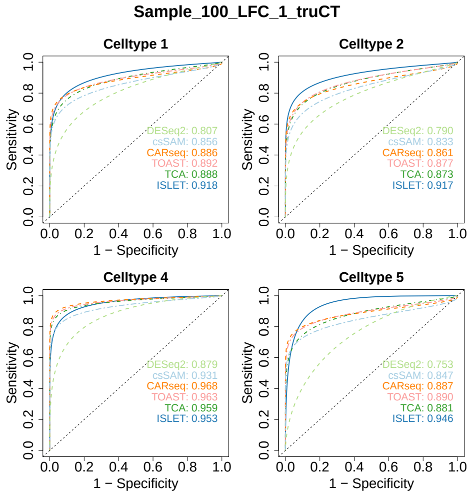

(c) LFC: 1

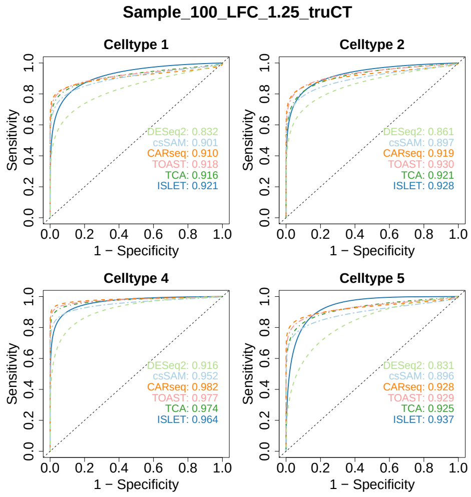

(d) LFC: 1.25

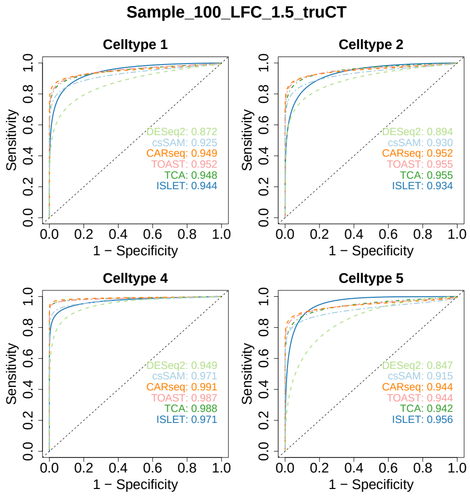

(e) LFC: 1.5

Figure S15: ROC curve plots for 4 cell types that contain csDEG, for sample size N=100 per group. (a)-(e) represent LFC 0.5 to 1.5.

1.3 Sensitivity versus FDR

The scatterplots are the sensitivity versus FDR, under an exhaustive combination of sample size and effect size, for all six methods.

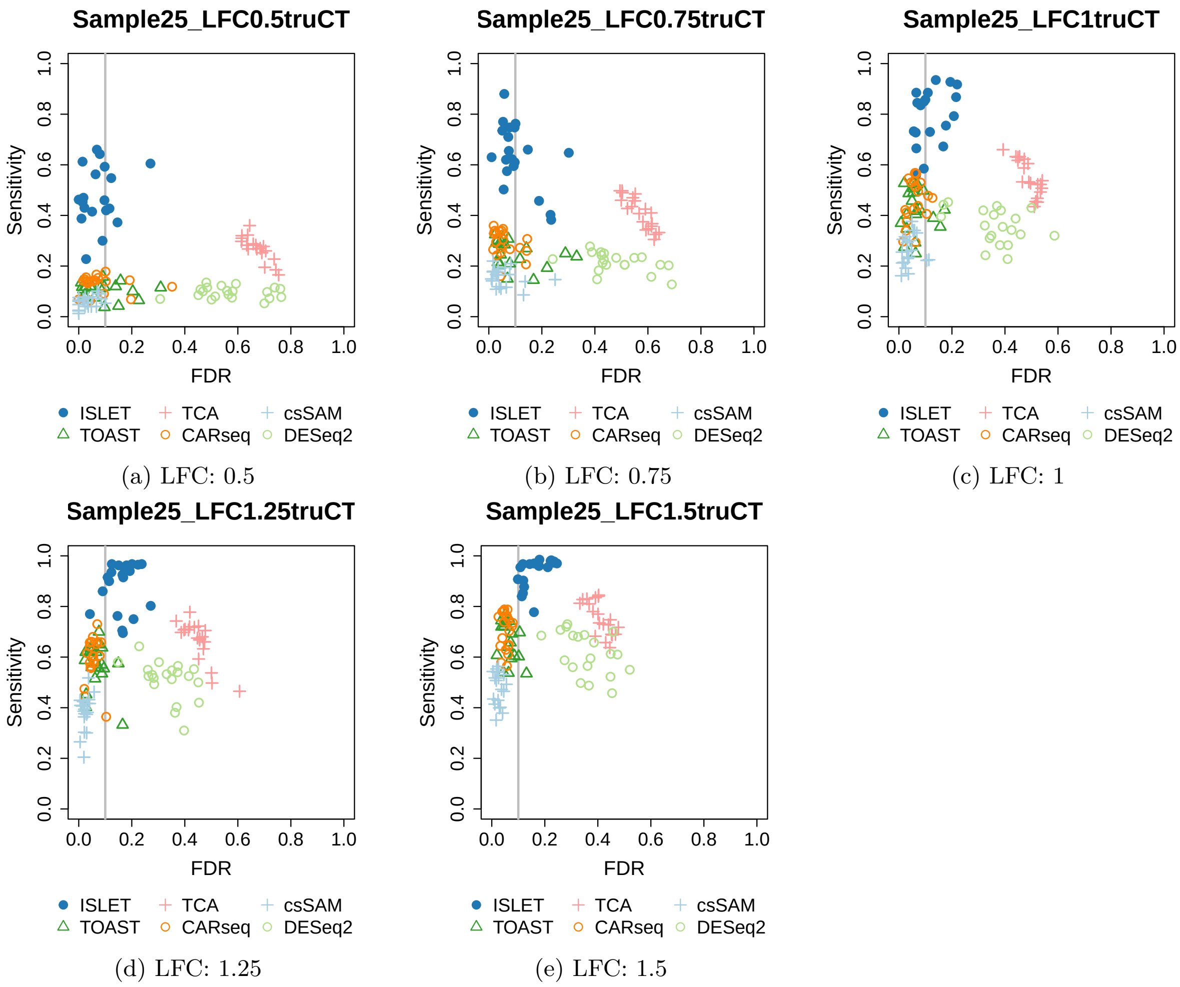

Figure S16: Sensitivity versus FDR plots for sample size N=25 per group. (a)-(e) represent LFC 0.5 to 1.5.

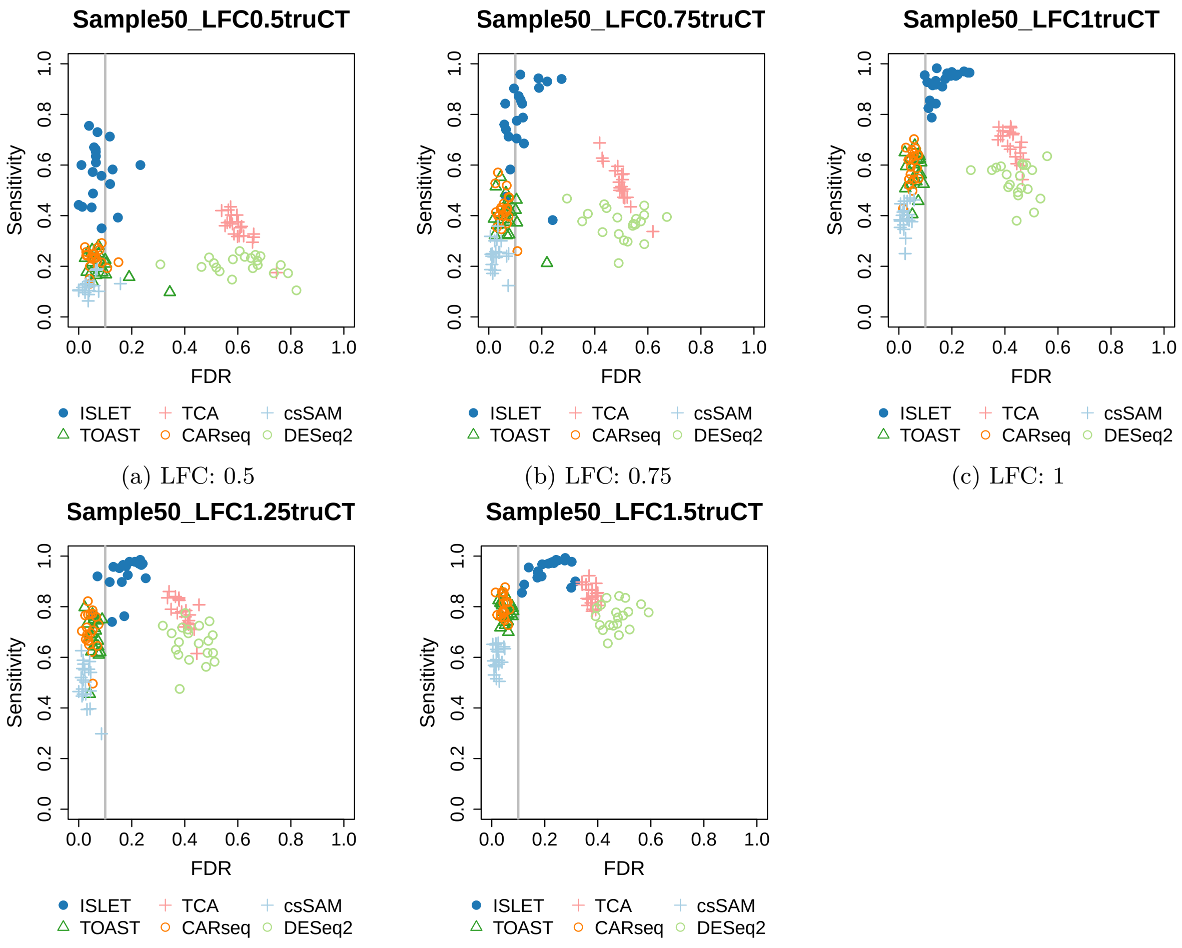

(a) LFC: 0.5

(b) LFC: 0.75

(c) LFC: 1

(d) LFC: 1.25

(e) LFC: 1.5

Figure S17: Sensitivity versus FDR plots for sample size N=50 per group. (a)-(e) represent LFC 0.5 to 1.5.

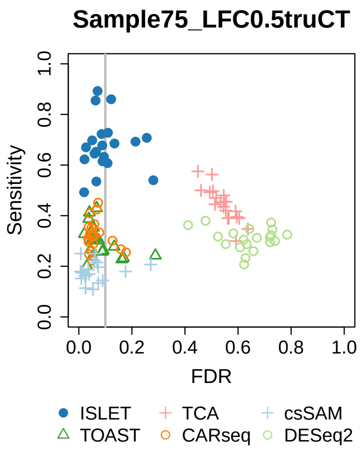

(a) LFC: 0.5

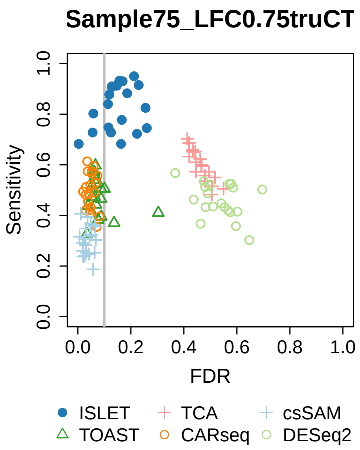

(b) LFC: 0.75

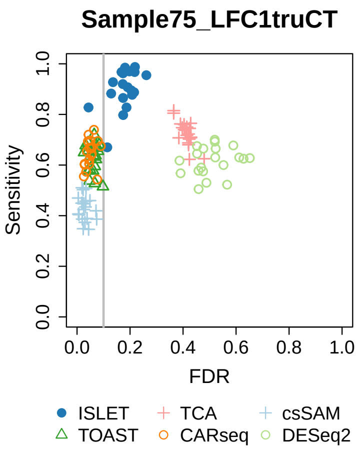

(c) LFC: 1

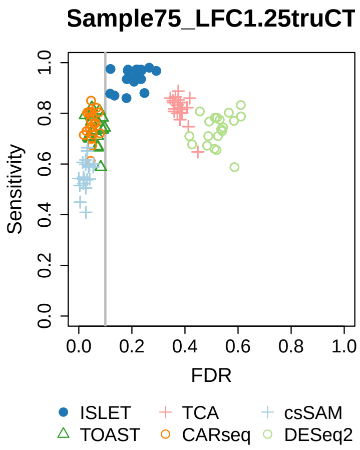

(d) LFC: 1.25

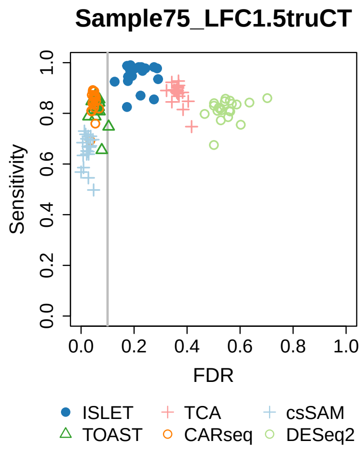

(e) LFC: 1.5

Figure S18: Sensitivity versus FDR plots for sample size N=75 per group. (a)-(e) represent LFC 0.5 to 1.5.

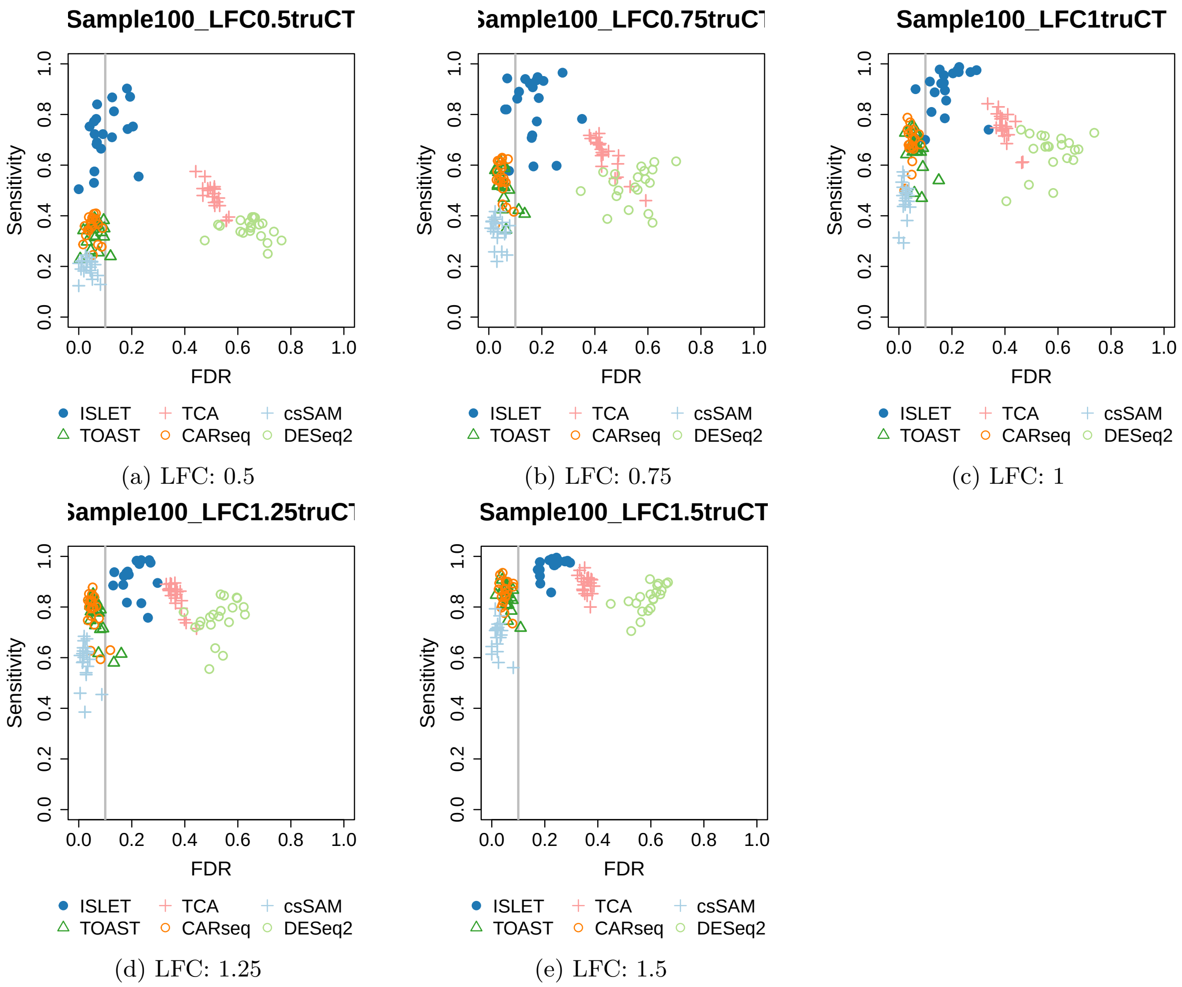

(a) LFC: 0.5 (b) LFC: 0.75 (c) LFC: 1

(d) LFC: 1.25 (e) LFC: 1.5

Figure S19: Sensitivity versus FDR plots for sample size N=100 per group. (a)-(e) represent LFC 0.5 to 1.5.

1.4 Sensitivity (cell-type-specific)

These are sensitivity along various effect sizes, at different sample sizes, for each cell type.

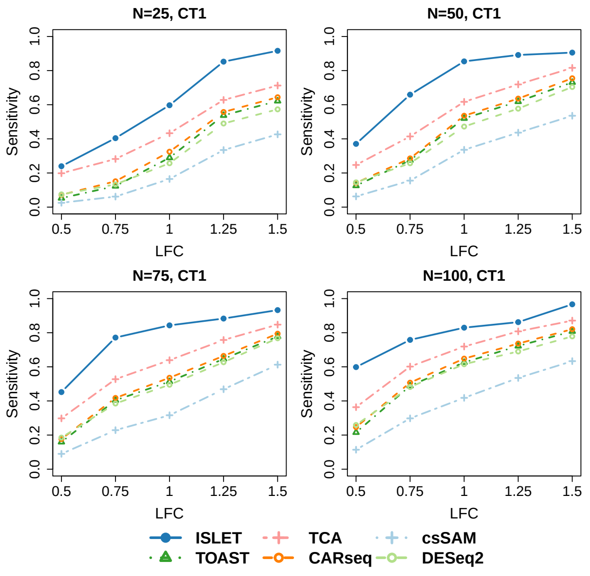

(a) Celltype1

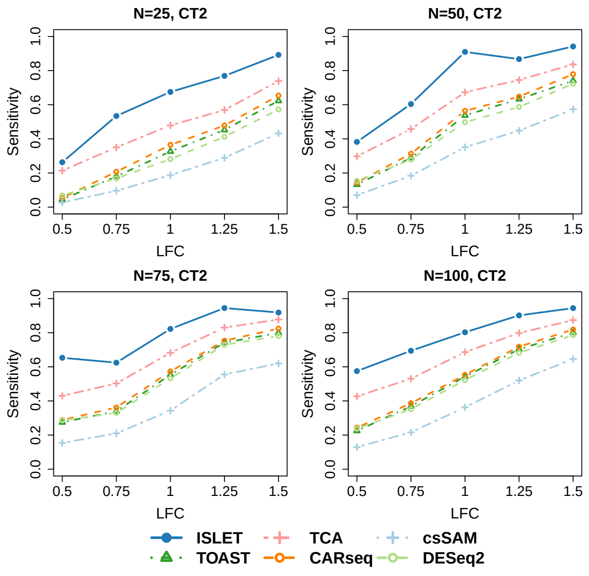

(b) Celltype2

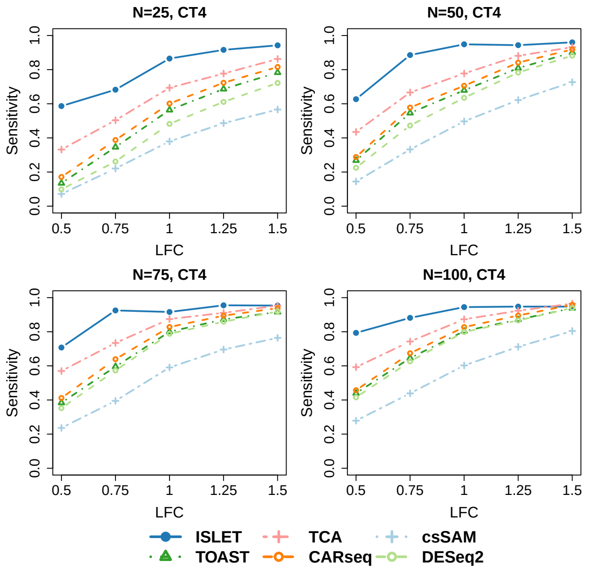

(c) Celltype4

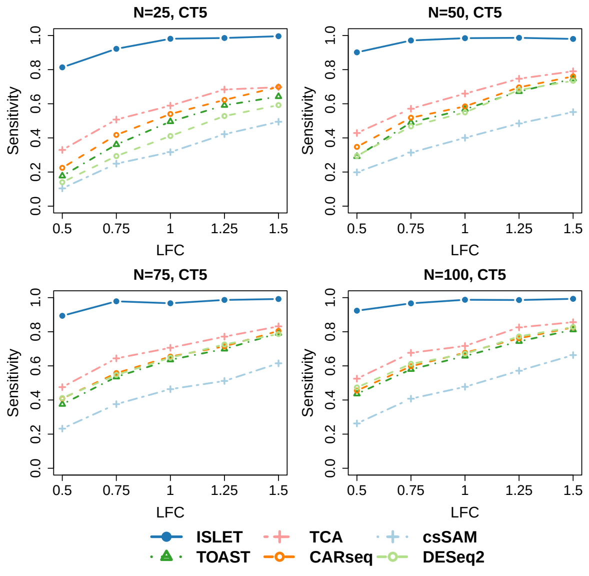

(d) Celltype5

Figure S20: Sensitivity shown along various effect sizes, for sample size N=25 to N=100 for each panel. (a)-(e) represent results over 4 different cell types that have csDEG.

1.5 Power comparison using true and estimated proportions.

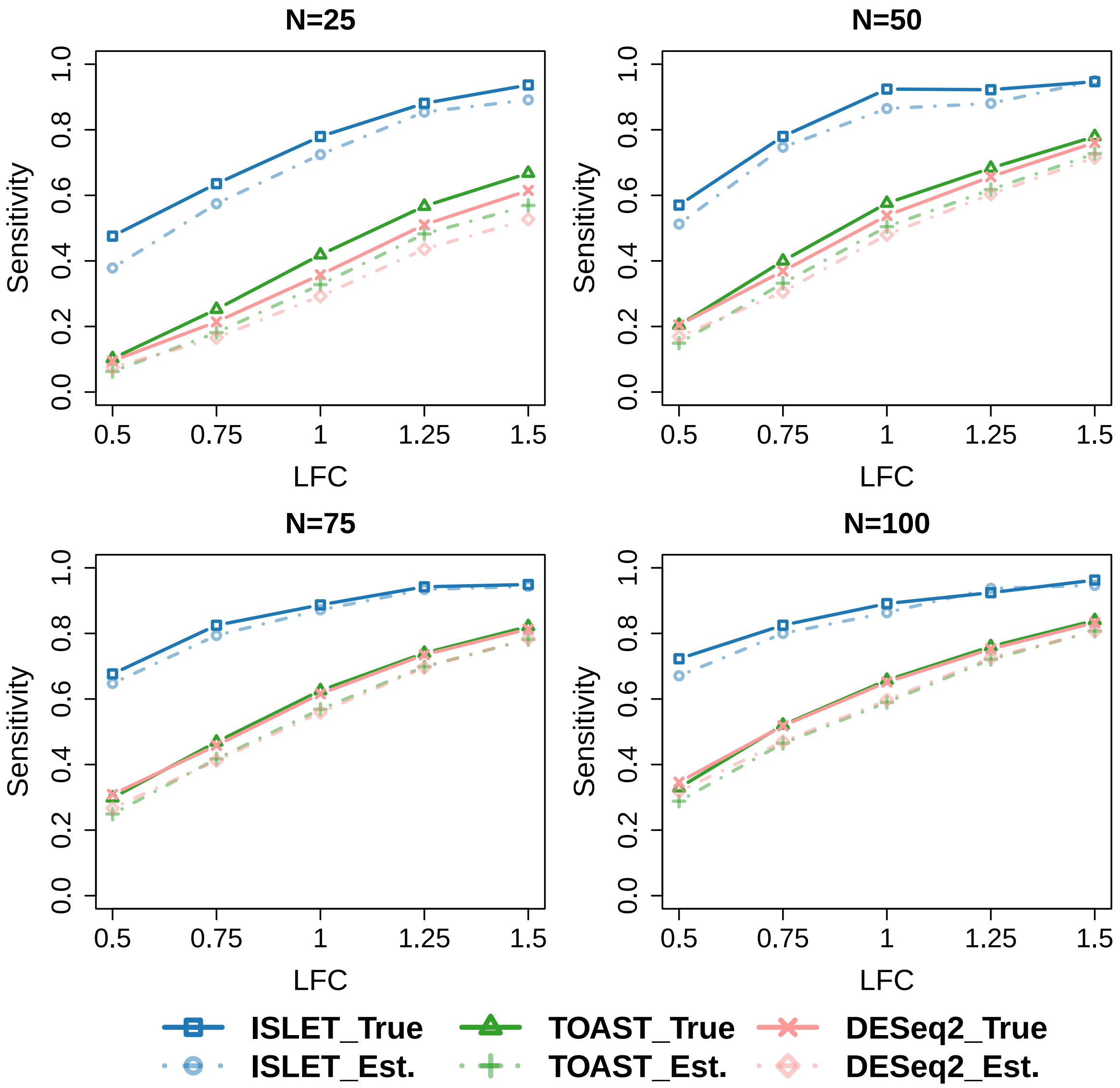

Figure S21: This plot compares the impact of two factors: (1) modeling choice and (2) the accuracy of cell type proportion estimates (true versus estimated), for ISLET, TOAST, and DESeq2. The analysis was conducted across different sample sizes, ranging from 25 to 100 subjects per group, with each panel representing a specific number of subjects. The gain of power using a proper modeling outweighs the imprecise cell type proportions.

1.6 Precision, ROC, FDR and power comparison using true and estimated proportions.

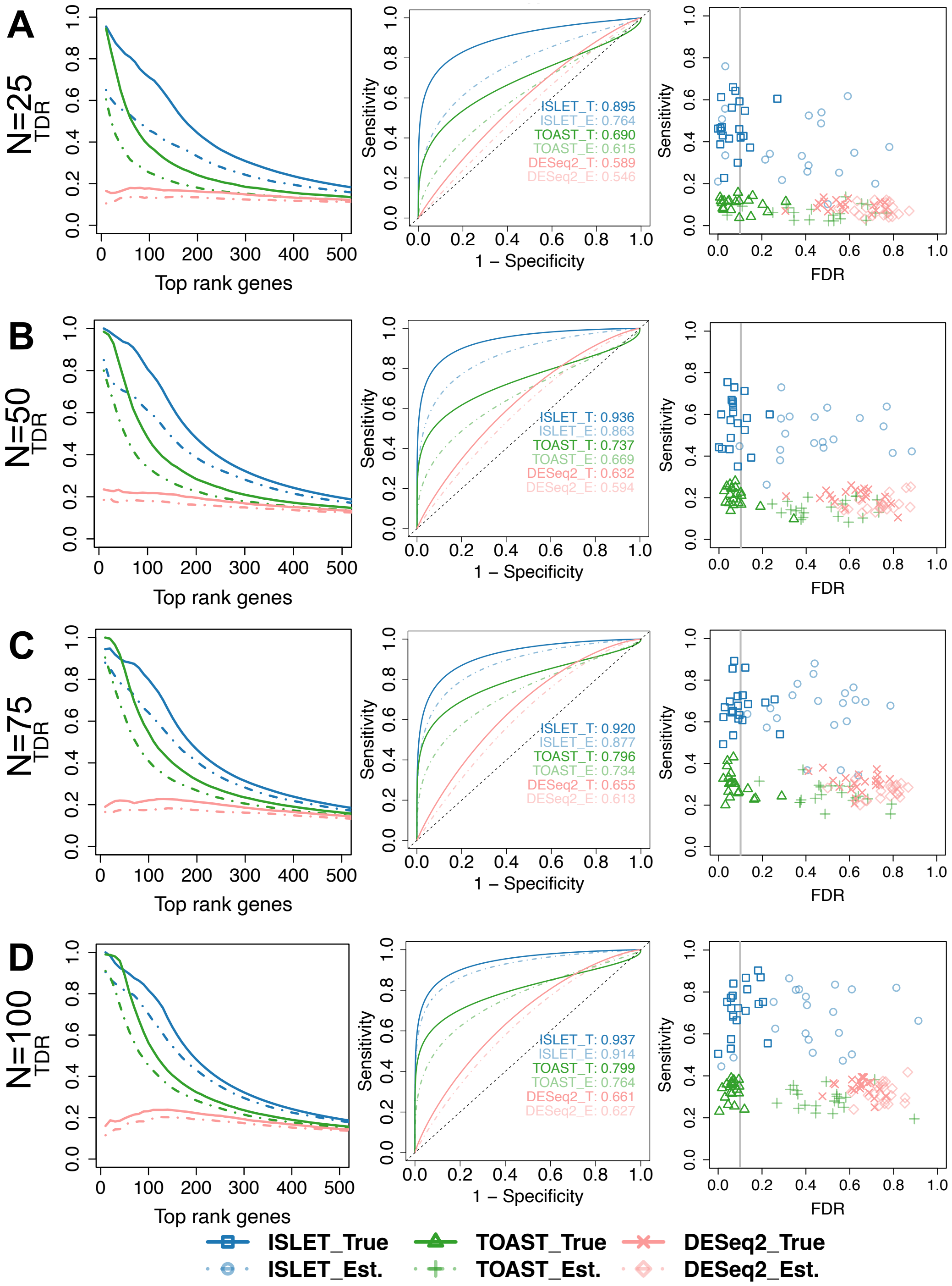

Figure S22: Overall impact comparison of two factors: (1) modeling choice and (2) the accuracy of cell type proportion estimates (true versus estimated), for ISLET, TOAST, and DESeq2. TDR, ROC, sensitivity versus FDR at different sample sizes are shown. LFC is fixed at 0.5 and the intended FDR level is at 0.1 in the third column panel.

1.7 Comparison under small sample size.

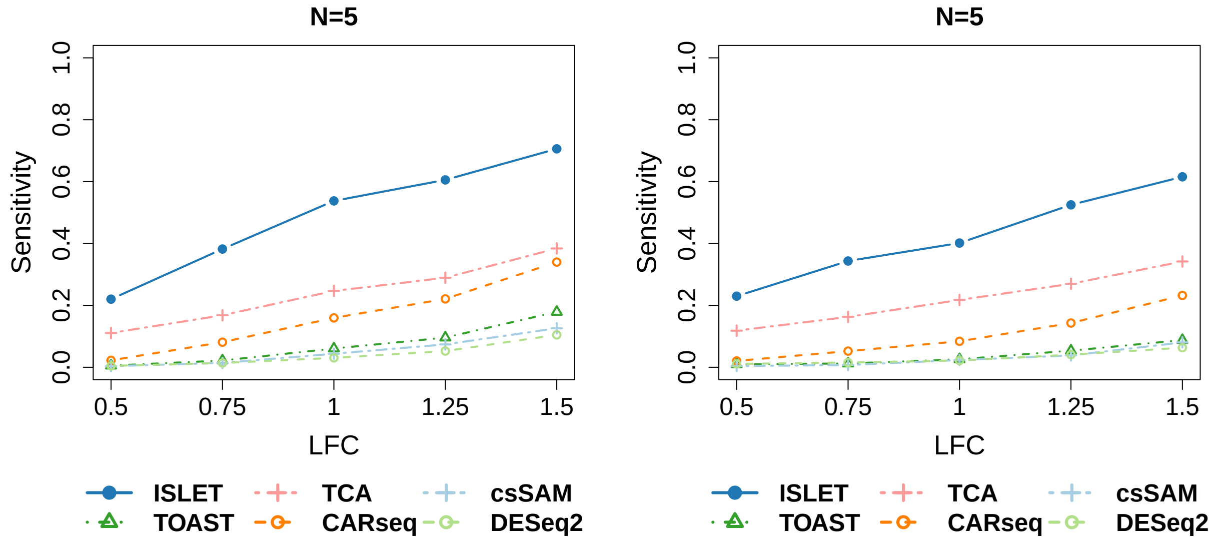

(a) True Cell Prop. (b) Est. Cell Prop.

Figure S23: Sensitivity shown along various effect sizes, for a small sample size (N=5 each group). The left and right panels respectively shows results obtained from the true and estimated cell proportion as inputs.

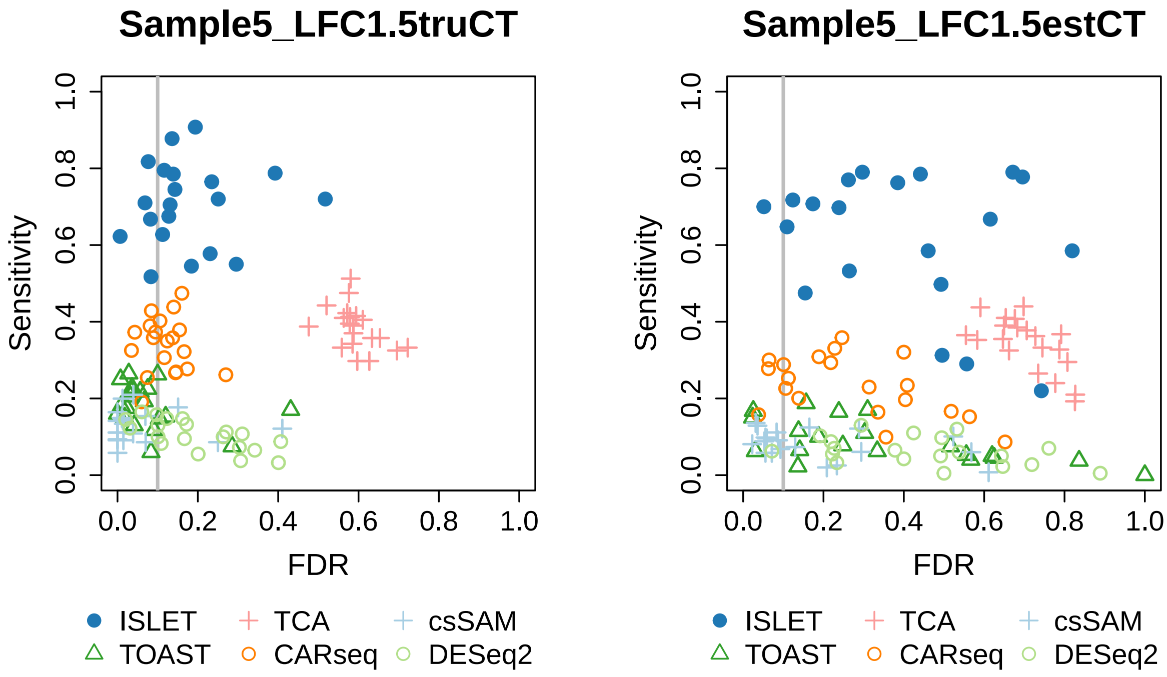

(a) True Cell Prop. (b) Est. Cell Prop.

Figure S24: Sensitivity versus FDR plots at a sample size (N=5 per group) and LFC set at 1.5. The left and right panels respectively shows results obtained from the true and estimated cell proportion as inputs.
