# Supplementary material for: ISLET: individual-specific reference panel recovery improves cell-type-specific inference
Source: Genome Biol. 2023 Jul 26;24:174. doi: 10.1186/s13059-023-03014-8 (PMC10373385; doi:10.1186/s13059-023-03014-8)
Supplement: Supplementary file 3 — Additional file 3. Simulation: ISLET slope test. [file 13059_2023_3014_MOESM3_ESM.pdf]

ISLET: individual-specific reference panel recovery improves cell-type-specific inference

Additional File 3

Simulation: ISLET slope test

Hao Feng*, Guanqun Meng, Tong Lin, Hemang Parikh, Yue Pan, Ziyi Li, Jeffrey Krischer and Qian Li*

Contents

1 ISLET slope test simulation result **2**

1.1 False Positive Rate (FPR) and Sensitivity by cell type 2

1.2 FPR, Sensitivity, False Discovery Rate (FDR) across cell types 4

1 ISLET slope test simulation result

1.1 False Positive Rate (FPR) and Sensitivity by cell type

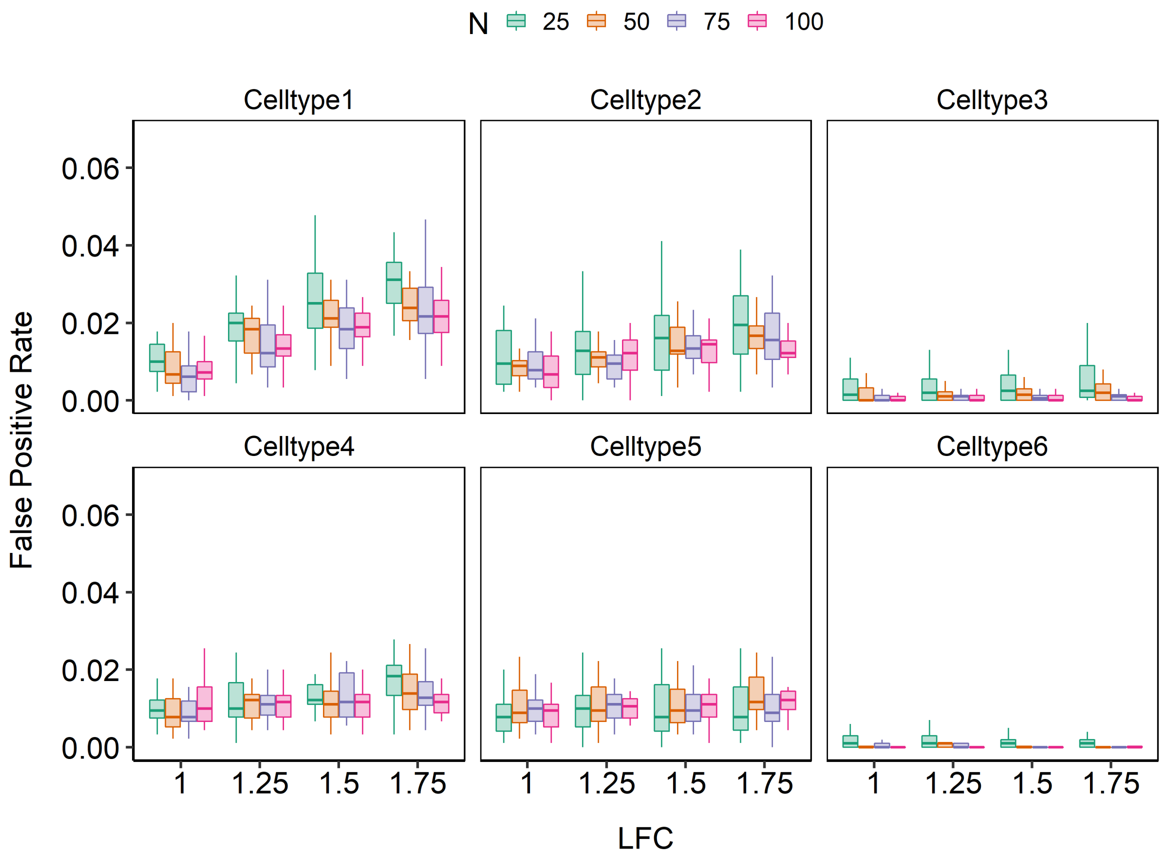

Figure S25: Boxplots of False Positive Rate comparing the LFC (1, 1.25, 1.5, 1.75) and sample size $N = 25, 50, 75, 100$ among 6 different cell types.

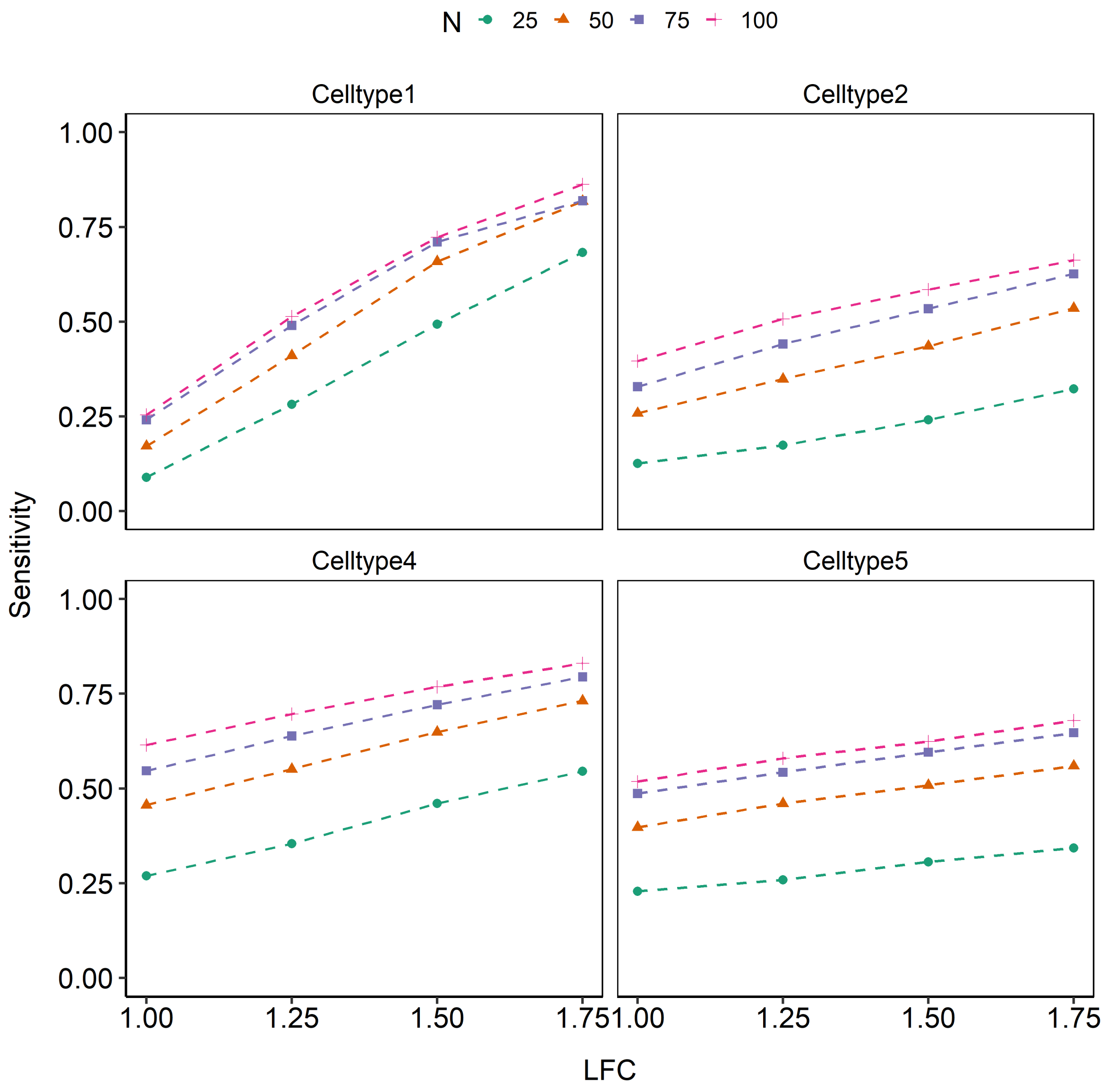

Figure S26: Sensitivity analysis of LFC (1, 1.25, 1.5, 1.75) and sample size $N = 25, 50, 75, 100$ among 4 different cell types with csDEG.

1.2 FPR, Sensitivity, False Discovery Rate (FDR) across cell types

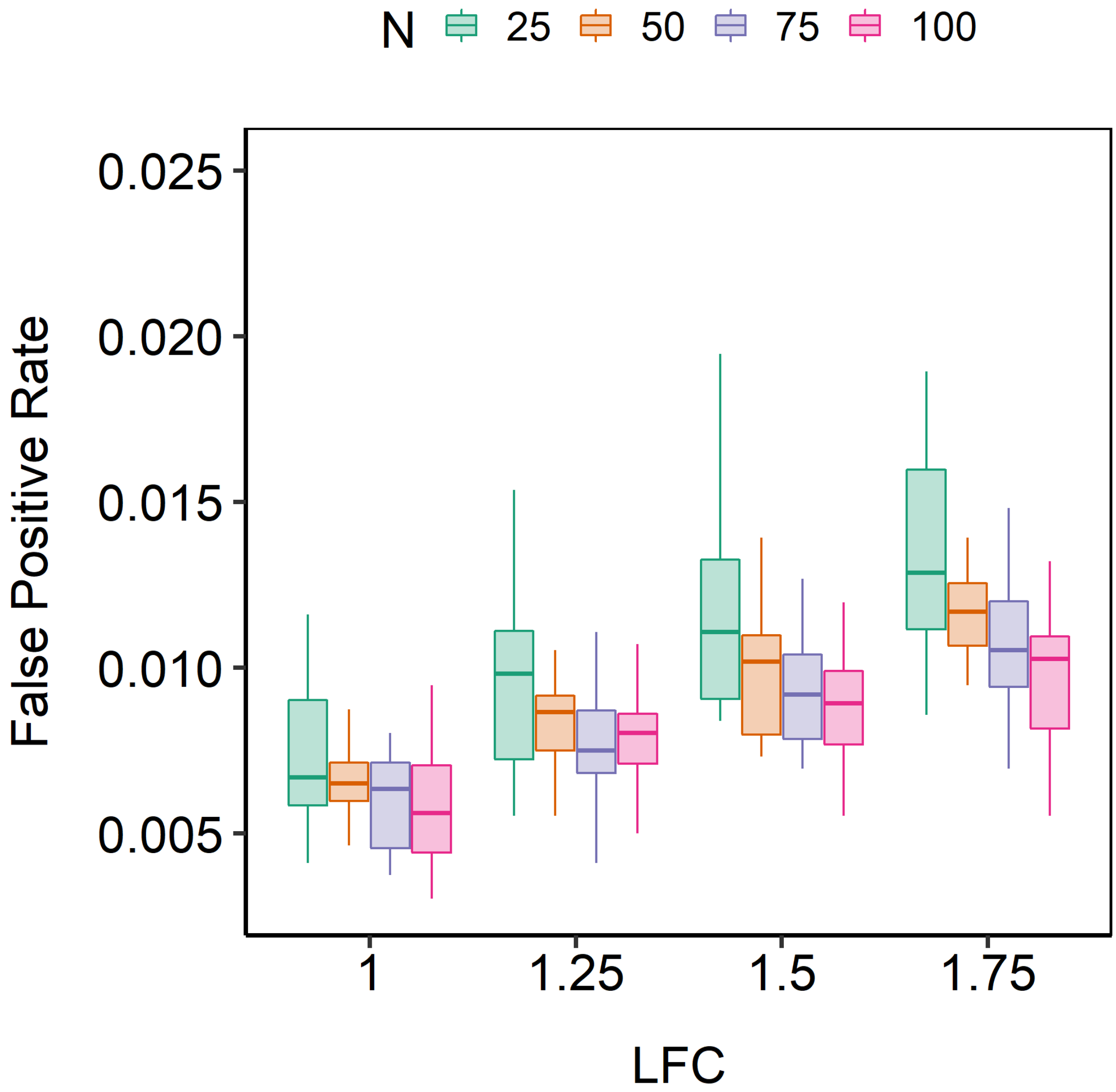

Figure S27: Boxplots of FPR comparing the LFC (1, 1.25, 1.5, 1.75) and sample size $N = 25, 50, 75, 100$ combining 6 different cell types.

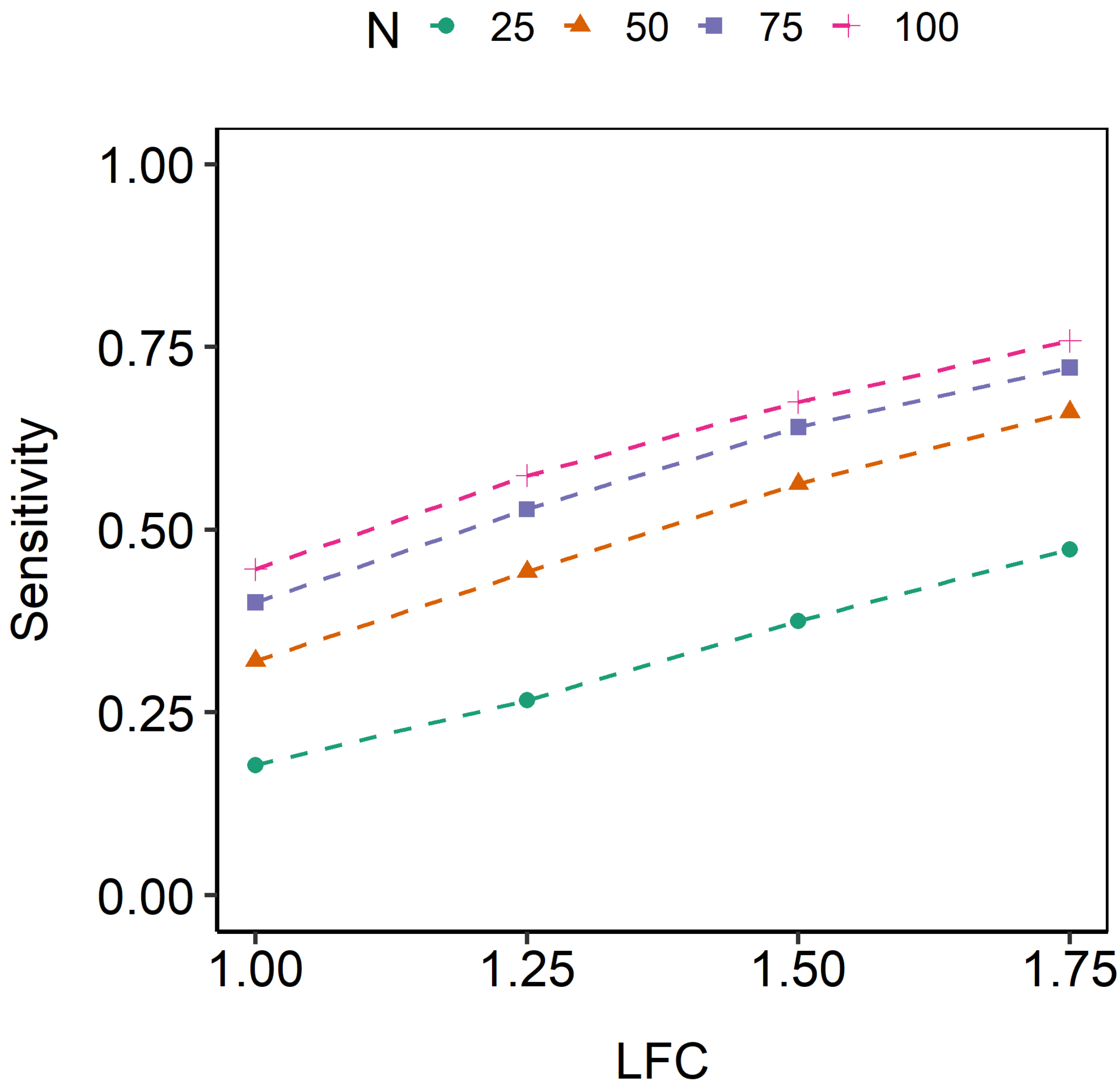

Figure S28: Sensitivity analysis of LFC (1, 1.25, 1.5, 1.75) and sample size $N = 25, 50, 75, 100$ combining 6 different cell types.

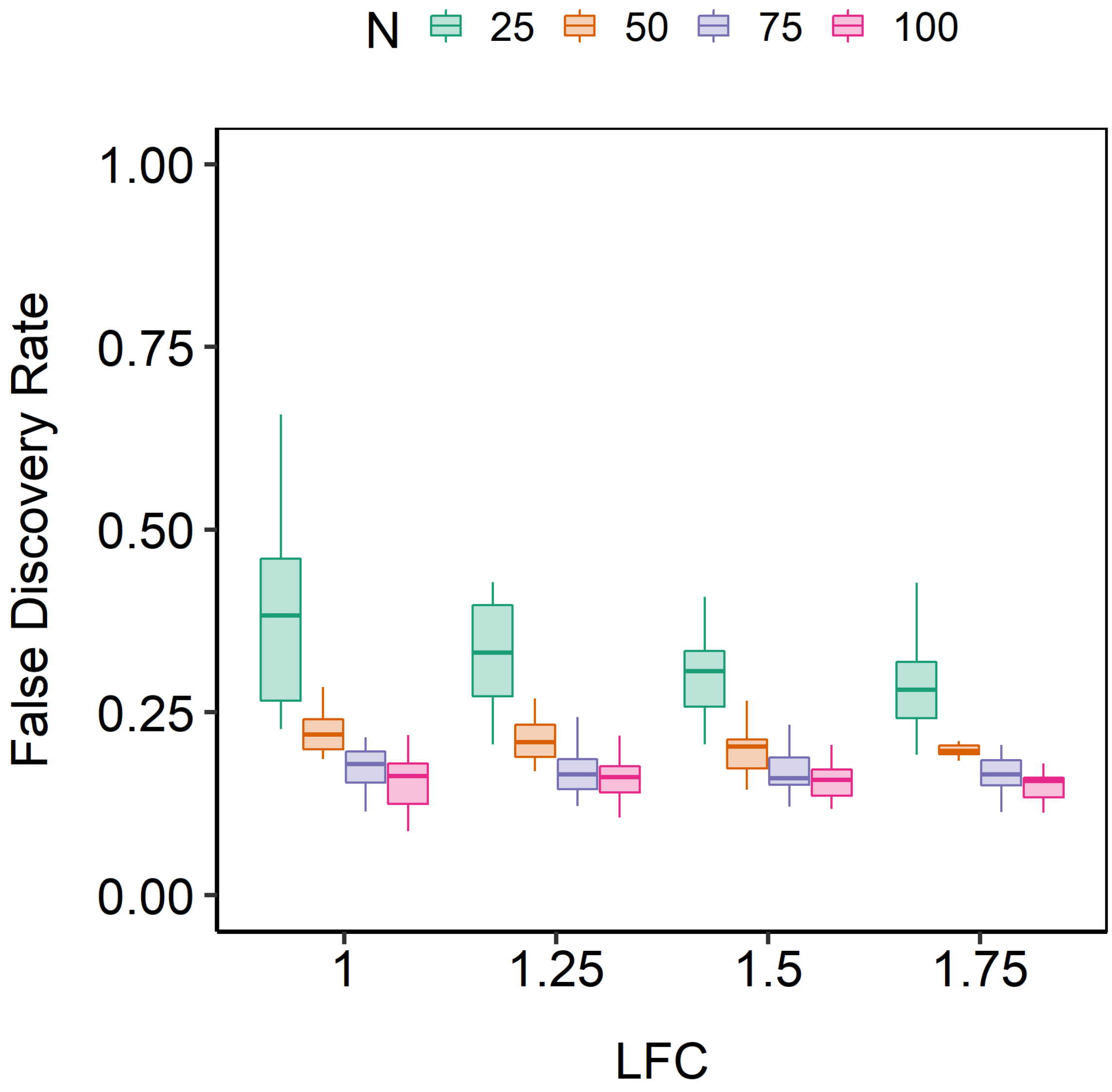

Figure S29: Boxplots of FDR comparing the LFC (1, 1.25, 1.5, 1.75) and sample size $N = 25, 50, 75, 100$ combining 6 different cell types.
