# Supplementary material for: ISLET: individual-specific reference panel recovery improves cell-type-specific inference
Source: Genome Biol. 2023 Jul 26;24:174. doi: 10.1186/s13059-023-03014-8 (PMC10373385; doi:10.1186/s13059-023-03014-8)
Supplement: Supplementary file 4 — Additional file 4. ISLET applications in real data. [file 13059_2023_3014_MOESM4_ESM.pdf]

ISLET: individual-specific reference panel recovery improves cell-type-specific inference

Additional File 4

ISLET applications in real data

Hao Feng*, Guanqun Meng, Tong Lin, Hemang Parikh, Yue Pan, Ziyi Li, Jeffrey Krischer and Qian Li*

Contents

1 TEDDY data analysis **2**

1.1 Overview of the TEDDY cohort and whole blood bulk RNA-seq data 2

1.2 Cell-type-specific Differentially Expressed Genes (csDEGs) detected by each method . . 3

1.3 Overlapped csDEGs in B-Cell and NK-Cell 8

1.4 Gene-wise Mean-Variance relation in TEDDY whole blood bulk RNA-seq data 12

2 PDBP data analysis **13**

1 TEDDY data analysis

1.1 Overview of the TEDDY cohort and whole blood bulk RNA-seq data

The TEDDY cohort screened and enrolled participants with susceptibility of T1D based on the Human Leukocyte Antigen (HLA) genotypes from six clinical centers in four countries (U.S., Finland, Germany, and Sweden). A total of 8,676 high-risk infants were enrolled from birth and followed every 3 months for blood sample collection and islet autoantibody (IAbs) measurement up to 4 years of age, then every 3-6 months based on autoantibody status until the age of 15 years or the diagnosis of T1D . The incidence of islet autoimmunity (IA) with persistently-confirmed IAbs in this prospective cohort started at the age of 9 months with a plateau between 1-2 years of age, depending on the type of autoantibodies. The participants who developed IA (cases) by May 31, 2012 and their matched controls (at 1:1 ratio) were selected for bulk RNA-seq transcriptome profiling, using the longitudinal whole blood samples prior to IAbs onset in each pair.

We used transcript per million (TPM) in this bulk RNA-seq data. The whole blood mRNA contains high levels of hemoglobin mRNAs (hgbRNA) due to the high-proportion of red blood cells, which can interfere with the counts of other genes [1]. Therefore, twelve hgbRNA genes (*HBA1, HBA2, HBB, HBBP1, HBD, HBE1, HBG1, HBG2, HBM, HBQ1, HBZ*, and *HBZP1*) were removed prior to TPM normalization [1]. Poor quality samples were first removed based on QC metrics from FastQC. The deconvolution of cell type proportions in the whole blood bulk RNA-seq samples was performed by AutoGeneS [2]. We excluded genes with mean TPM-normalized counts less than 1 and used two scRNA-seq datasets as AutoGeneS input. The first dataset was from the whole blood and whole bone marrow scRNA-seq data generated by the 10X Genomics and BD Rhapsody platforms [3], and the second was PBMC data generated on the 10X Genomics platform [4].

The TEDDY RNA-seq samples were prepared using Illumina's TruSeq Stranded mRNA Sample Prep Kit. Sequences were generated on the Illumina HiSeq4000 platform with paired-end 2 x 101 bp reads with a targeted 50 million reads per sample by the Broad Institute, Cambridge, MA. The raw sequences were aligned by using the Gencode Genome Reference Consortium Build 38 (GRCh38.p12) Release 31 reference genome and comprehensive gene annotation. Reads were aligned using the STAR aligner [5] (version 2.6.1d) based on the Trans-Omics for Precision Medicine (TOPMed) GTEx RNA-seq analysis pipeline. Ribosomal RNA (rRNA) was identified and removed using bbsplit (version 38.86) (https://sourceforge.net/projects/bbmap/) prior to alignment using Ensembl rRNA (Ensembl Archive Release 97) as reference. The gene expression was quantified by RNA-SeQC (version 2.1.0) [6]. The RNA quality per sample was evaluated further by the qSVA score [7]. The samples with qSVA score 3×standard deviation less than mean were removed from our analysis.

1.2 Cell-type-specific Differentially Expressed Genes (csDEGs) detected by each method

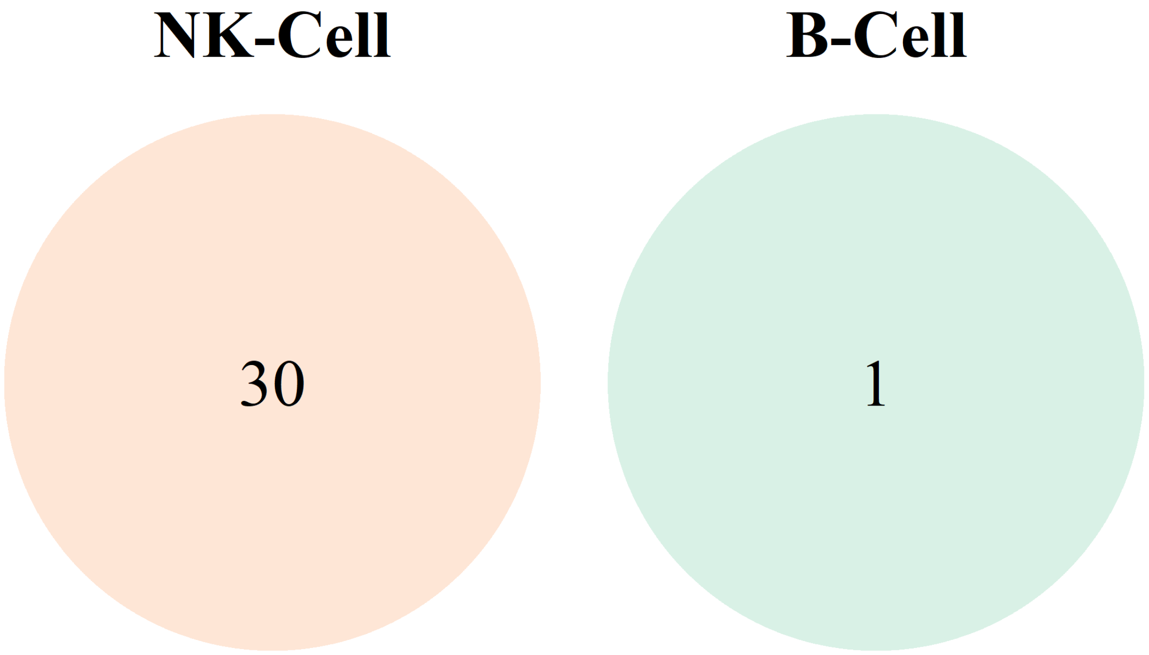

Figure S30: csDEGs called by ISLET

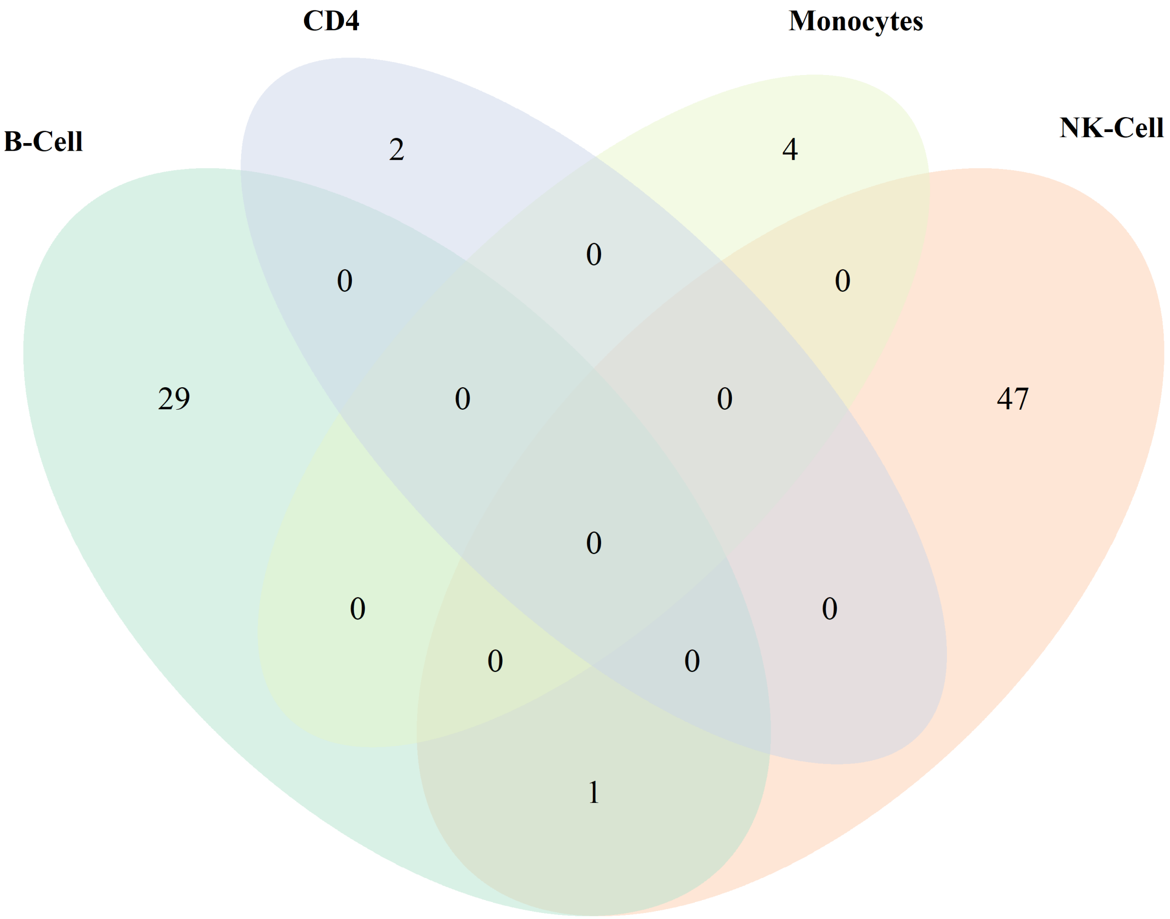

Figure S31: csDEGs called by TOAST

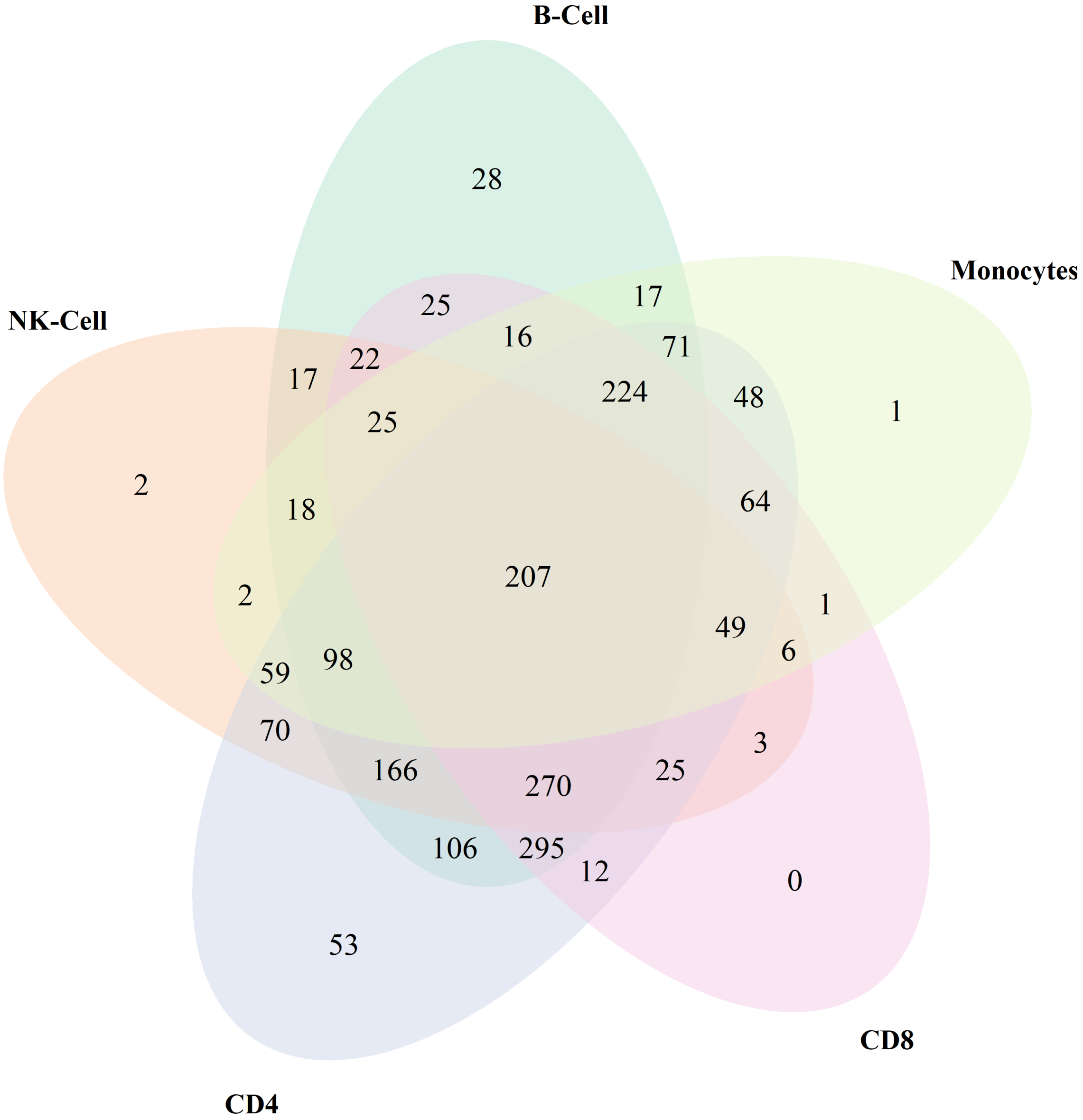

Figure S32: csDEGs called by TCA

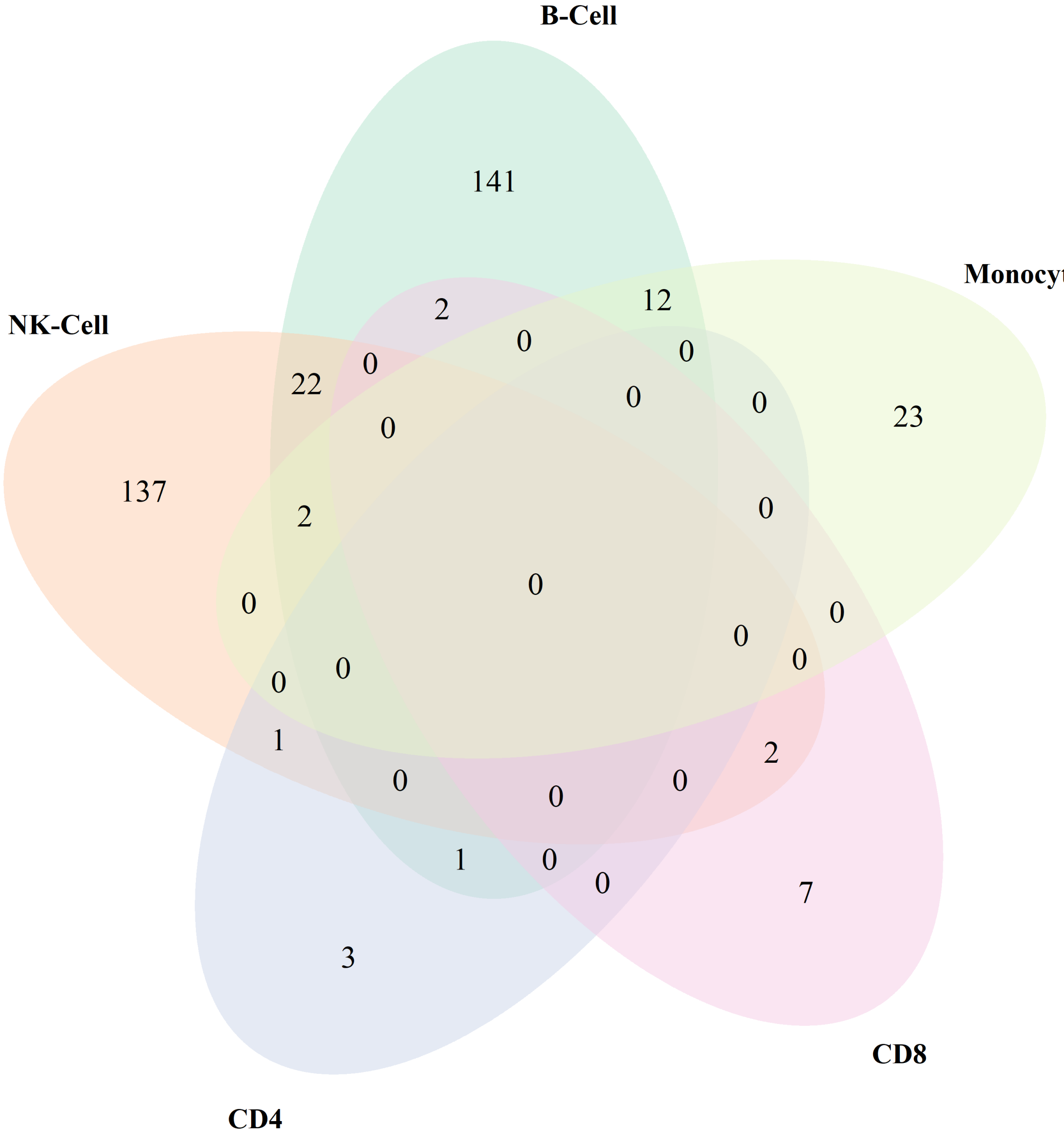

Figure S33: csDEGs called by DESeq2

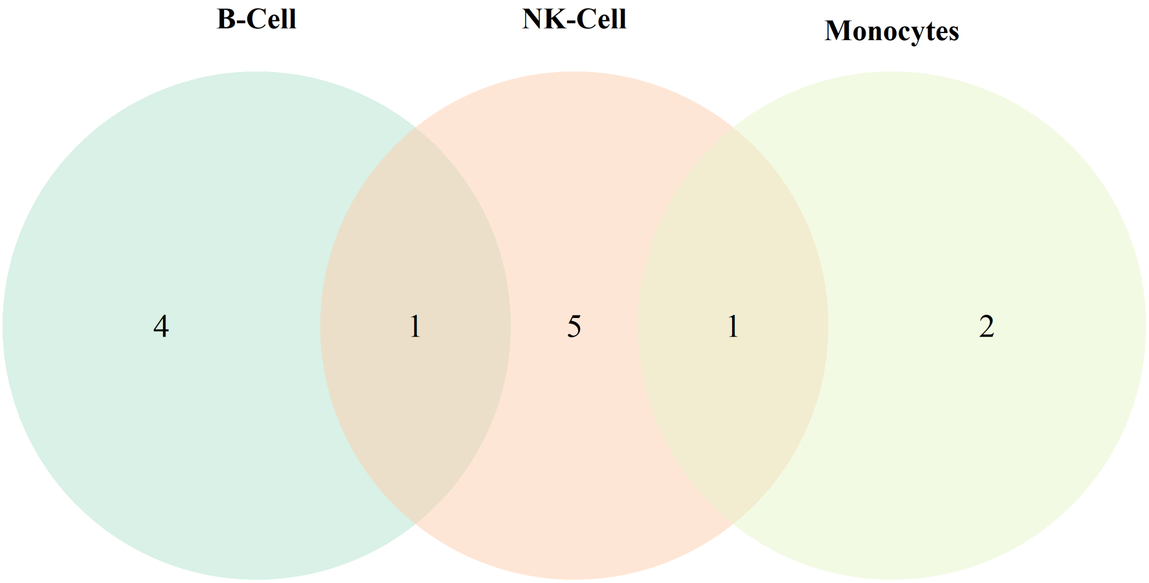

Figure S34: csDEGs called by CARseq

1.3 Overlapped csDEGs in B-Cell and NK-Cell

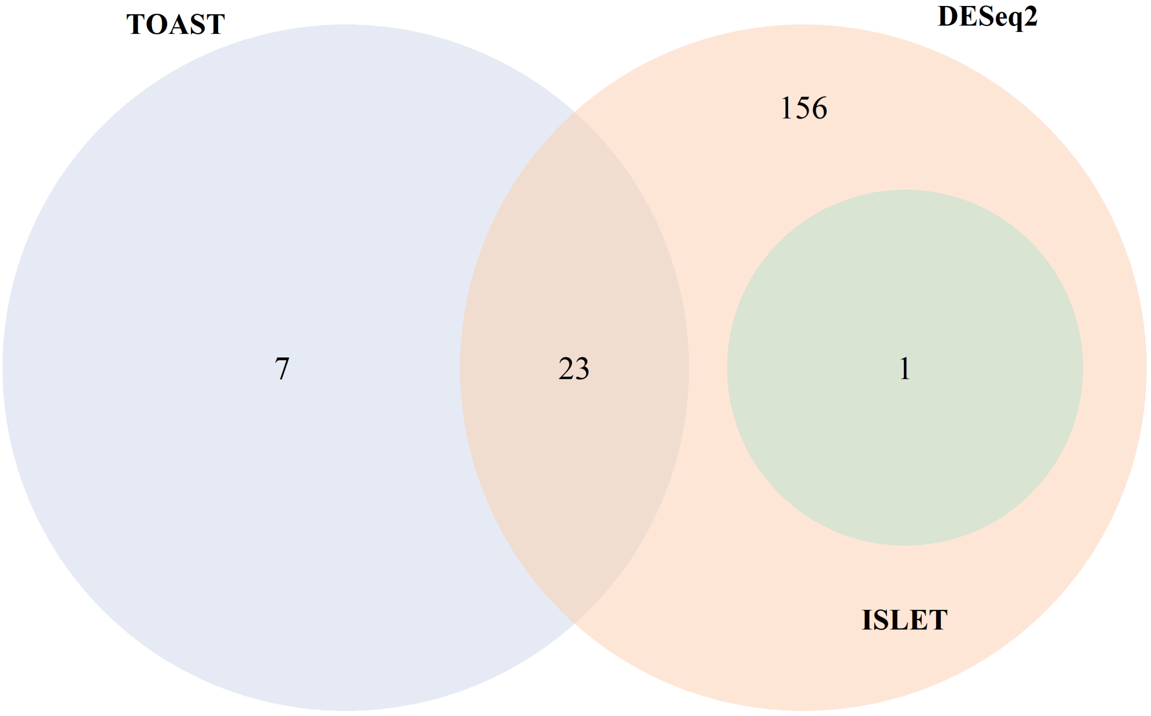

Figure S35: B-cell-specific DEGs called by ISLET, TOAST, DESeq2

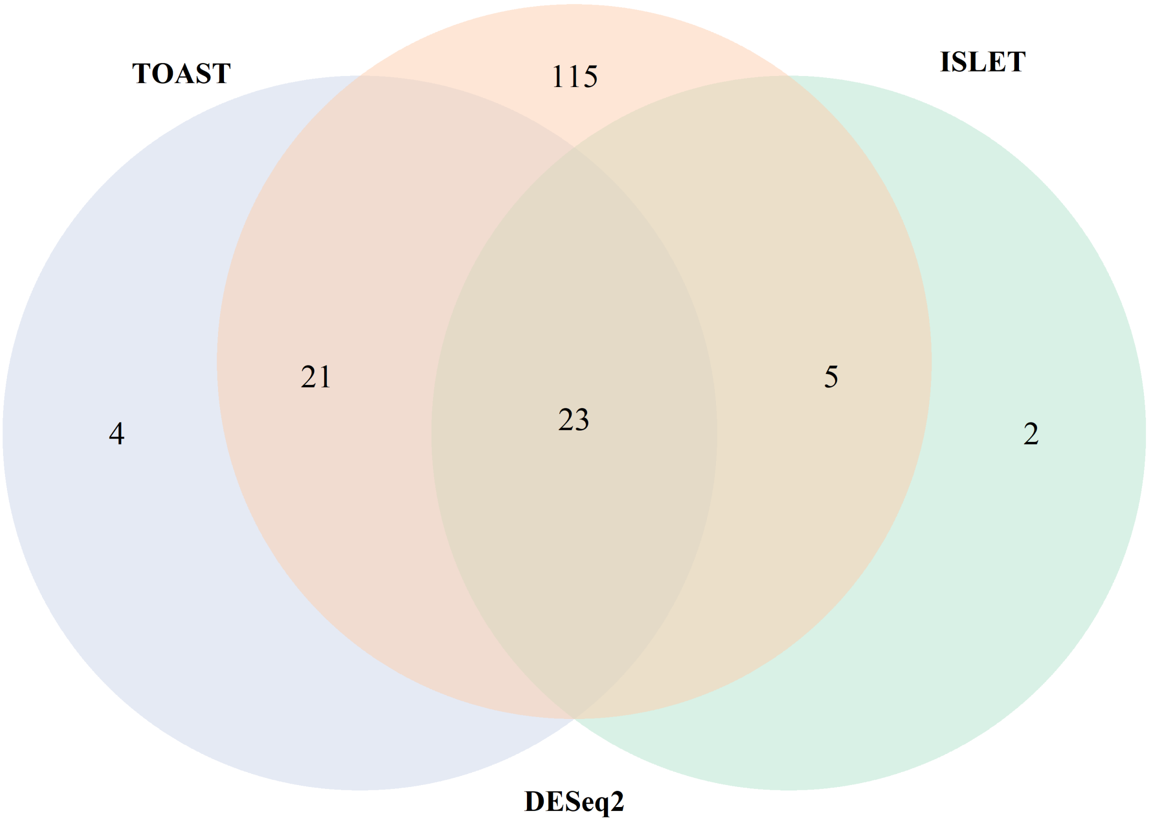

Figure S36: NK-cell-specific DEGs called by ISLET, TOAST, DESeq2

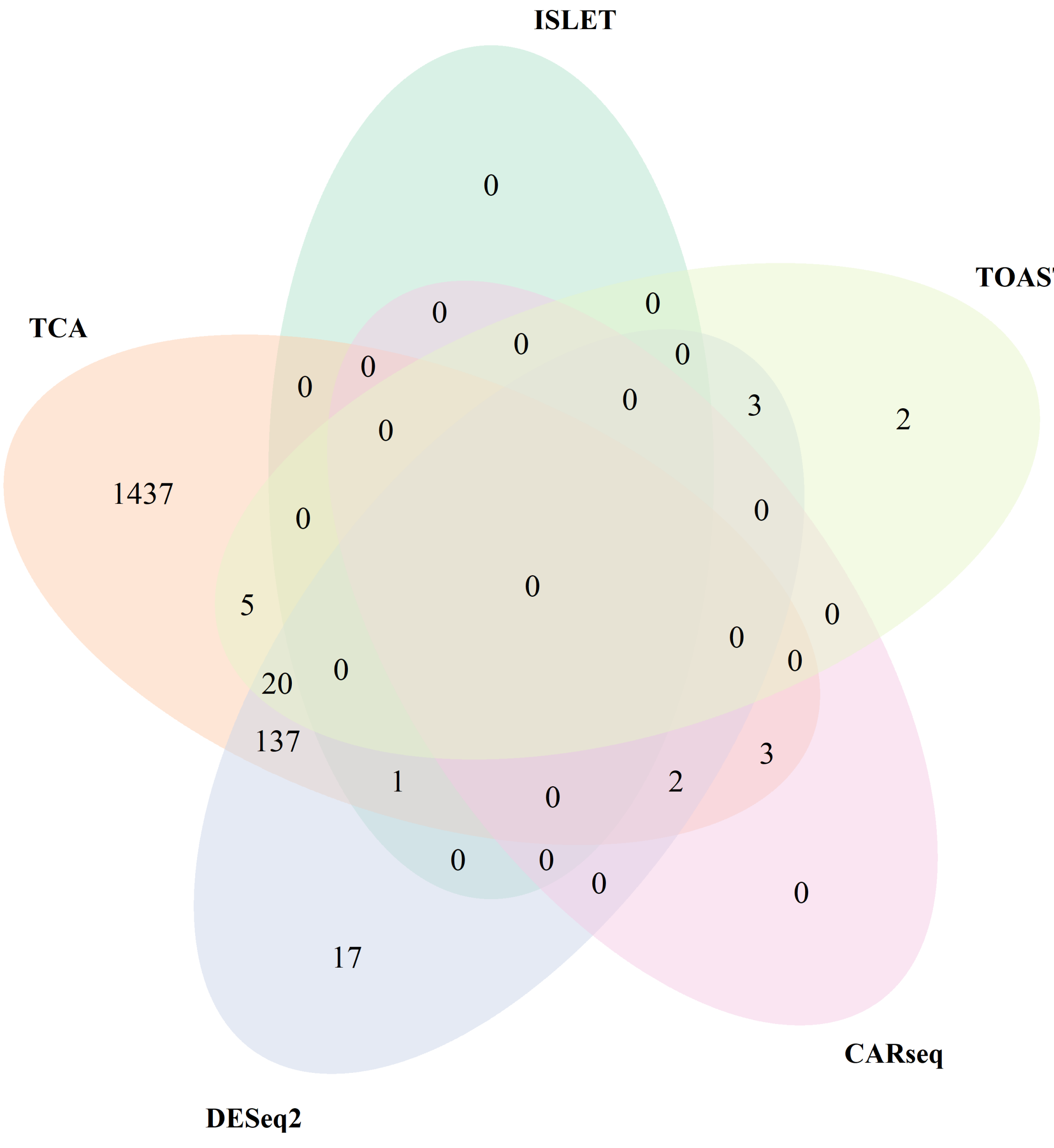

Figure S37: B-cell-specific DEGs called by ISLET, TOAST, DESeq2, CARseq, TCA

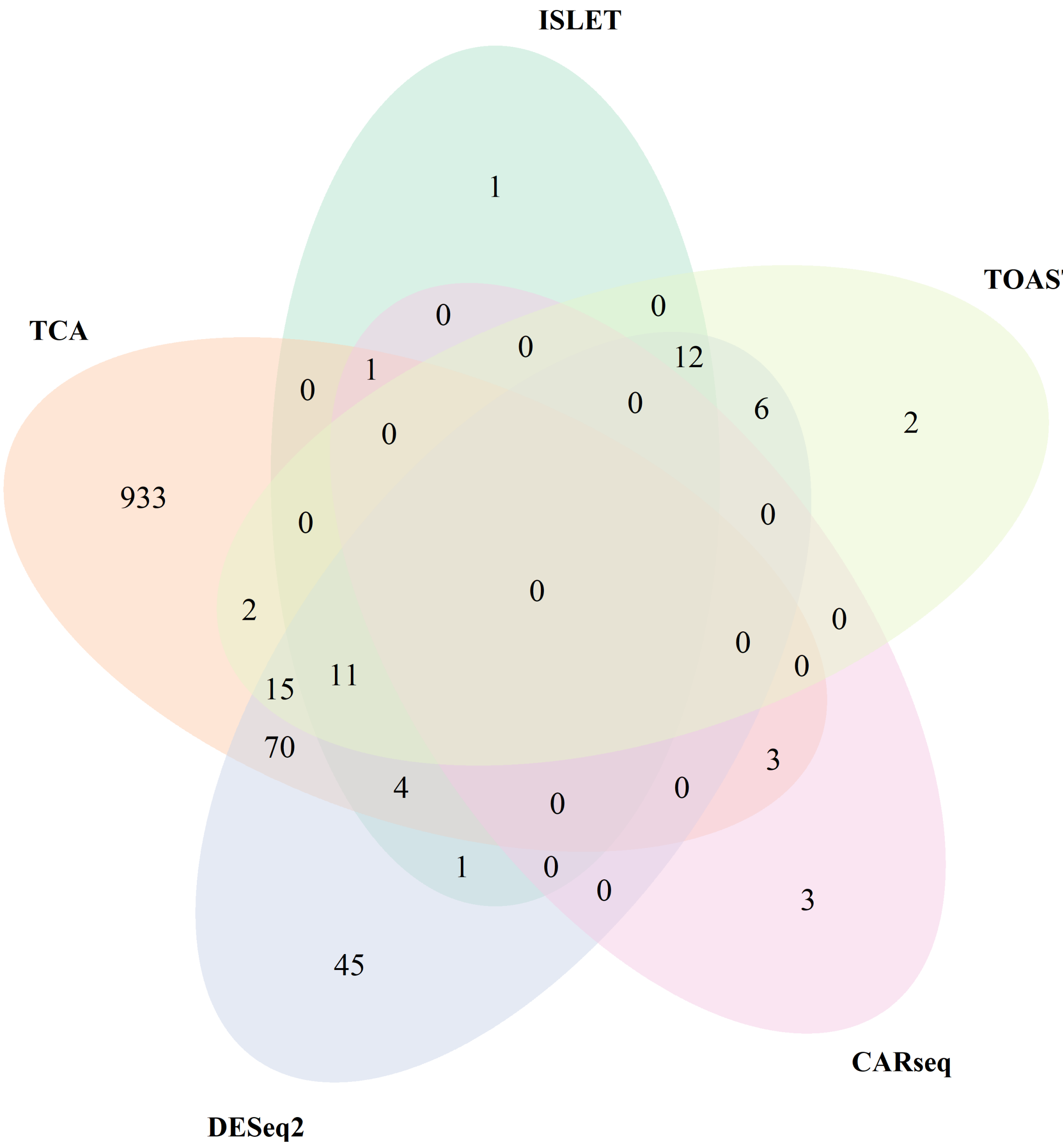

Figure S38: NK-cell-specific DEGs called by ISLET, TOAST, DESeq2, CARseq, TCA

1.4 Gene-wise Mean-Variance relation in TEDDY whole blood bulk RNA-seq data

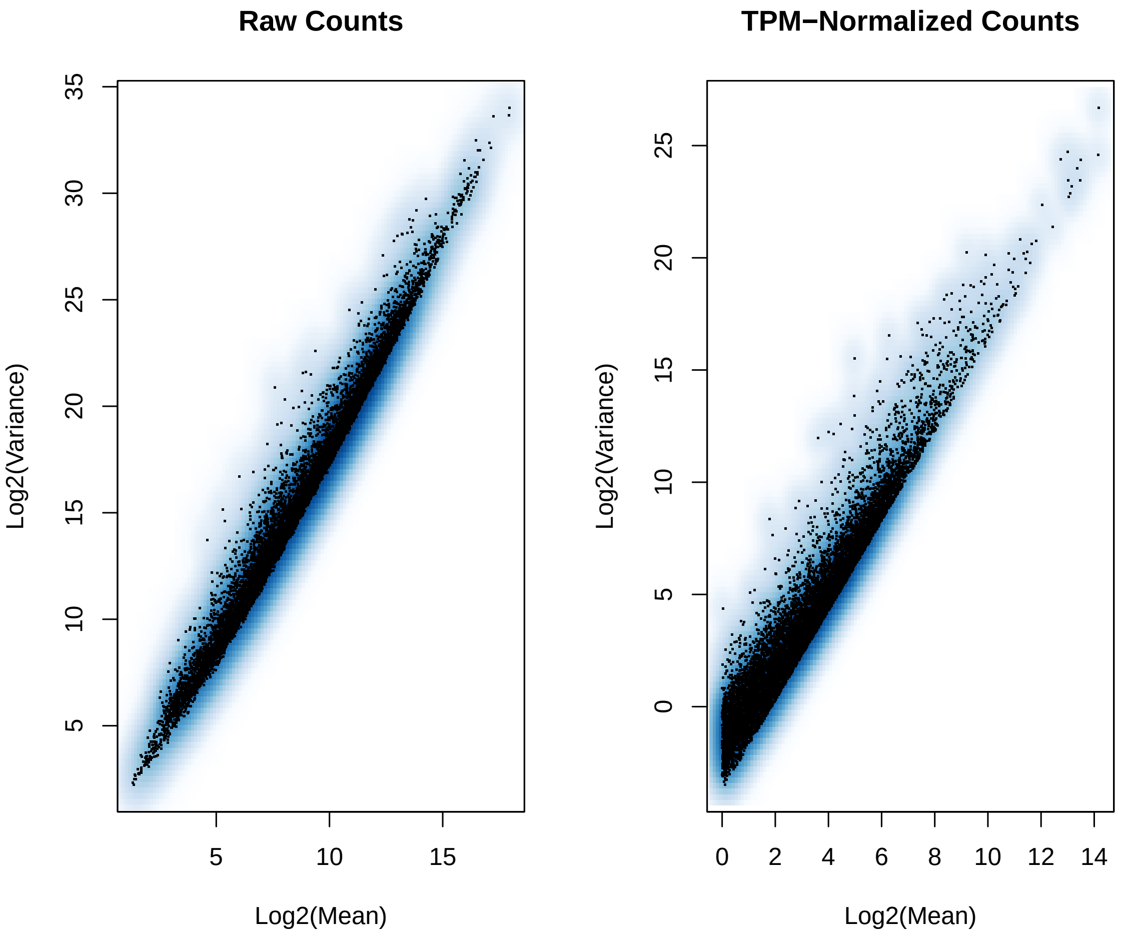

Figure S39: Distribution of mean-variance per gene in integer raw counts (left panel) and Transcript Per Million (TPM)-normalized counts (right panel) in TEDDY data

2 PDBP data analysis

The results listed below display the ISLET testing results for csDEGs on Parkinson's Disease Biomarkers Program (PDBP) dataset, for a group of 572 study participants, with longitudinal observations for 399 cases and 173 controls. Bulk transcriptome data from whole blood were deconvoluted and ISLET was adopted to identify csDEGs within each cell type.

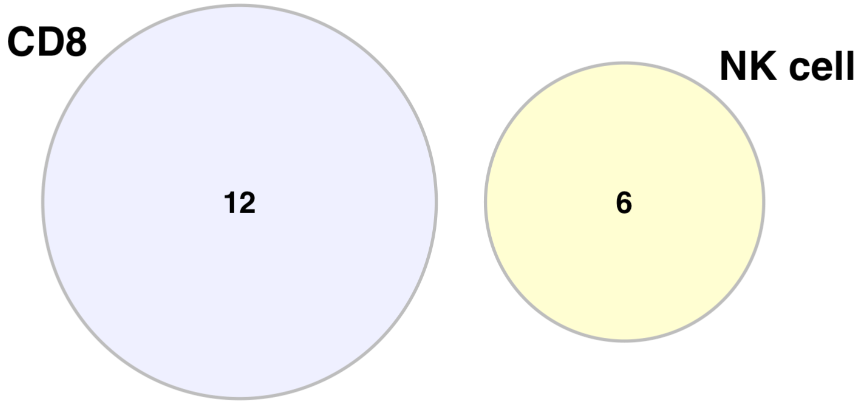

Figure S40: Parkinson disease csDEGs detected by ISLET.

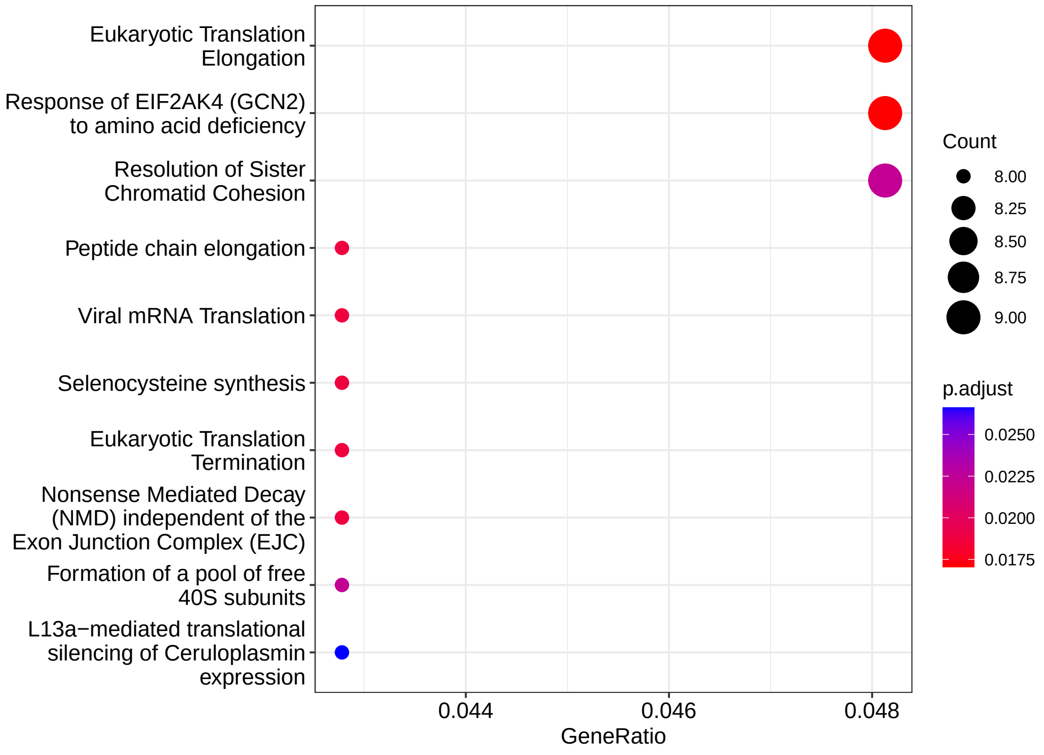

Figure S41: Gene Ontology (GO) analysis of all csDEGs combined from all cell types to explore pathways that are potentially relevant to PD.

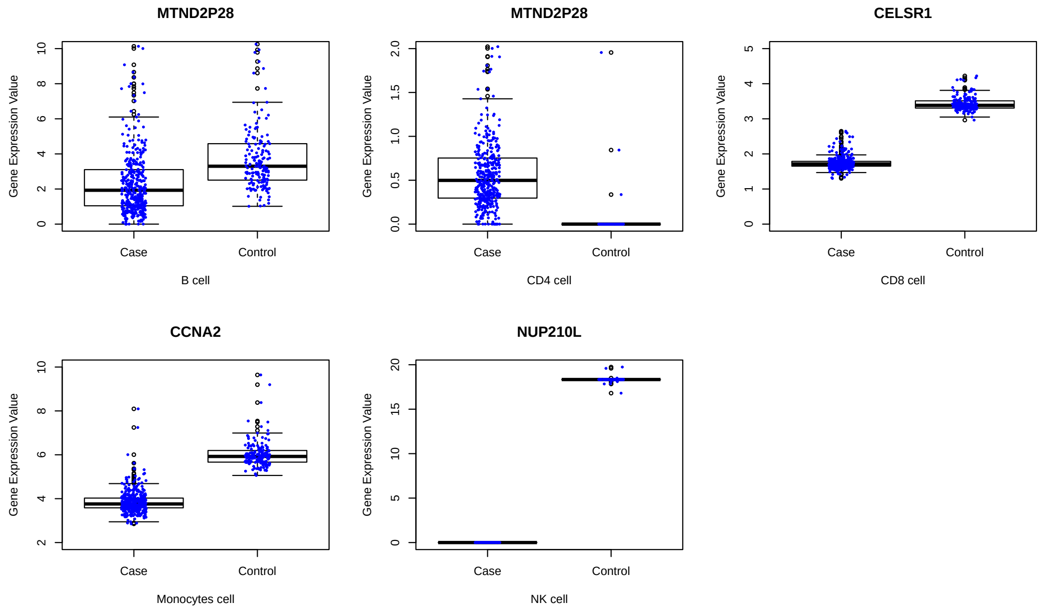

Figure S42: Recovered individual-specific deconvoluted expression for five exemplary genes reported in previous PD studies. Gene name is listed on the top and cell type name is listed at the bottom in each panel.

References

[1] Harrington, C.A., Fei, S.S., Minnier, J., Carbone, L., Searles, R., Davis, B.A., Ogle, K., Planck, S.R., Rosenbaum, J.T., Choi, D.: Rna-seq of human whole blood: Evaluation of globin rna depletion on ribo-zero library method. Scientific reports **10**(1), 1–12 (2020)

[2] Aliee, H., Theis, F.J.: Autogenes: Automatic gene selection using multi-objective optimization for rna-seq deconvolution. Cell Systems **12**(7), 706–715 (2021)

[3] Qi, J., D'Souza, D., Dawson, T., Geanon, D., Stefanos, H., Marvin, R., Walker, L., Rahman, A.H.: Multimodal single-cell characterization of the human granulocyte lineage. bioRxiv (2021)

[4] Hao, Y., Hao, S., Andersen-Nissen, E., Mauck III, W.M., Zheng, S., Butler, A., Lee, M.J., Wilk, A.J., Darby, C., Zager, M., *et al.*: Integrated analysis of multimodal single-cell data. Cell **184**(13), 3573–3587 (2021)

[5] Dobin, A., Davis, C.A., Schlesinger, F., Drenkow, J., Zaleski, C., Jha, S., Batut, P., Chaisson, M., Gingeras, T.R.: Star: ultrafast universal rna-seq aligner. Bioinformatics **29**(1), 15–21 (2013)

[6] DeLuca, D.S., Levin, J.Z., Sivachenko, A., Fennell, T., Nazaire, M.-D., Williams, C., Reich, M., Winckler, W., Getz, G.: Rna-seqc: Rna-seq metrics for quality control and process optimization. Bioinformatics **28**(11), 1530–1532 (2012)

[7] Jaffe, A.E., Tao, R., Norris, A.L., Kealhofer, M., Nellore, A., Shin, J.H., Kim, D., Jia, Y., Hyde, T.M., Kleinman, J.E., *et al.*: qsva framework for rna quality correction in differential expression analysis. Proceedings of the National Academy of Sciences **114**(27), 7130–7135 (2017)
