# Supplementary material for: ISLET: individual-specific reference panel recovery improves cell-type-specific inference
Source: Genome Biol. 2023 Jul 26;24:174. doi: 10.1186/s13059-023-03014-8 (PMC10373385; doi:10.1186/s13059-023-03014-8)
Supplement: Supplementary file 5 — Additional file 5. Appendix. [file 13059_2023_3014_MOESM5_ESM.pdf]

ISLET: individual-specific reference panel recovery improves cell-type-specific inference

Additional File 5

Appendix

Hao Feng*, Guanqun Meng, Tong Lin, Hemang Parikh, Yue Pan, Ziyi Li, Jeffrey Krischer and Qian Li*

Contents

1 Heterogeneity between and within subjects in pure PBMC gene expression **2**

2 The model in matrix and EM algorithm **3**

1 Heterogeneity between and within subjects in pure PBMC gene expression

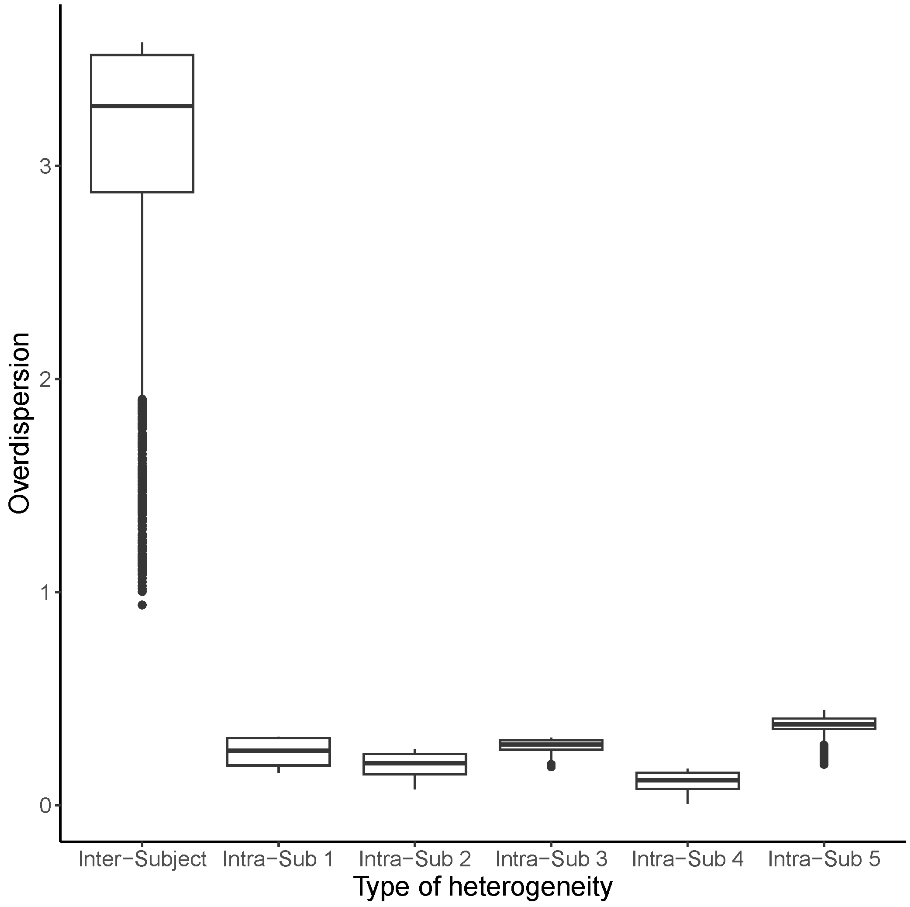

Figure S43: Gene-specific overdispersion in B cells between control subjects or within each subject.

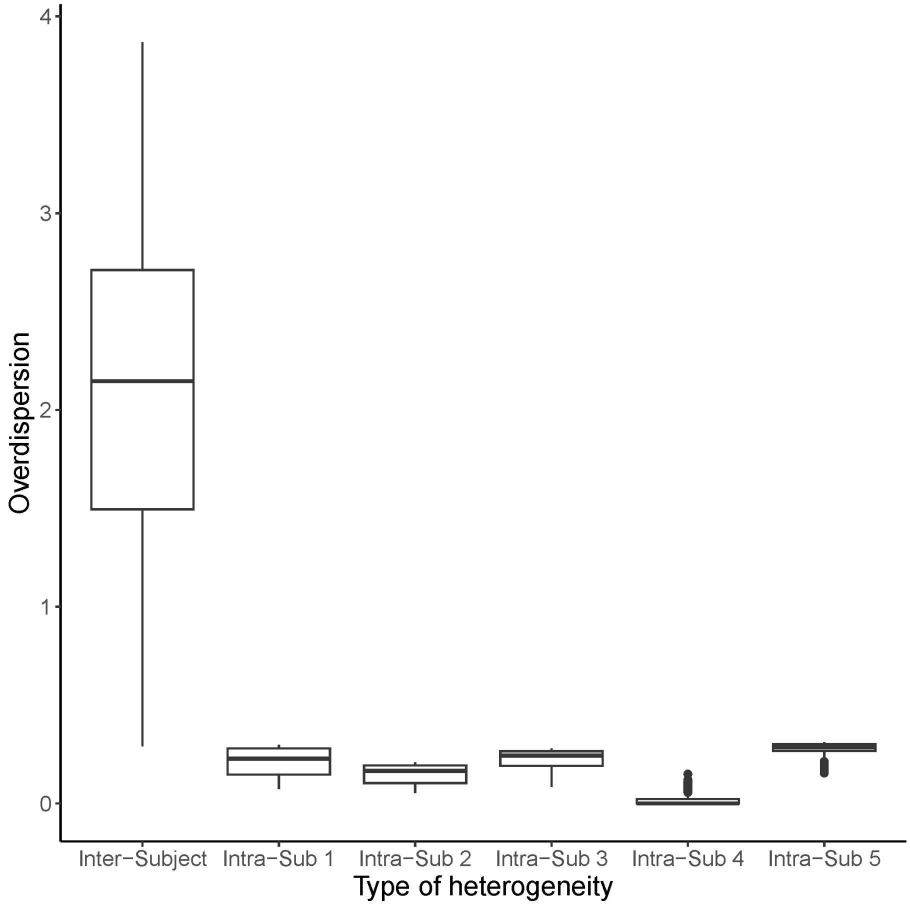

Figure S44: Gene-specific overdispersion in CD4+ T cells between control subjects or within each subject.

2 The model in matrix and EM algorithm

We use y_{jt} to represent the observed gene expression for subject j at time-point t. The dependent variable vector is thus $\boldsymbol{y}$, where $\boldsymbol{y} = (y_{11}, y_{12}, \cdots, y_{1T_1}, \cdots, y_{J1}, y_{J2}, \cdots, y_{JT_J})'$, with length $N = \sum_{j=1}^{J} T_j$. Denote $Q = JK$. Here, $\boldsymbol{X}$ and $\boldsymbol{A}$ are the design matrices for the fixed-effect $\boldsymbol{\beta}$ and random-effect $\boldsymbol{u}$, respectively, where $\boldsymbol{\beta} = (m_1, m_2, \cdots, m_K, \beta_1, \beta_2, \cdots, \beta_K)'$, and $\boldsymbol{u} = (u_{11}, u_{21}, \cdots, u_{J1}, u_{12}, u_{22}, \cdots, u_{J2}, \cdots, u_{1K}, u_{2K}, \cdots, u_{JK})'$. The linear model in matrix form for

all subjects is

$$\boldsymbol{y} = \boldsymbol{X\beta} + \boldsymbol{Au} + \boldsymbol{\varepsilon} \tag{A.1}$$

where

$$\boldsymbol{X} = \begin{pmatrix} \theta_{111} & \theta_{112} & ... & \theta_{11K} & z_1\theta_{111} & z_1\theta_{112} & ... & z_1\theta_{11K} \\ \theta_{121} & \theta_{122} & ... & \theta_{12K} & z_1\theta_{121} & z_1\theta_{122} & ... & z_1\theta_{12K} \\ ... & ... & ... & ... & ... & ... & ... & ... \\ \theta_{1T_11} & \theta_{1T_12} & ... & \theta_{1T_1K} & z_1\theta_{1T_11} & z_1\theta_{1T_12} & ... & z_1\theta_{1T_1K} \\ ... & ... & ... & ... & ... & ... & ... & ... \\ \theta_{J11} & \theta_{J12} & ... & \theta_{J1K} & z_J\theta_{J11} & z_J\theta_{J12} & ... & z_J\theta_{J1K} \\ \theta_{J21} & \theta_{J22} & ... & \theta_{J2K} & z_J\theta_{J21} & z_J\theta_{J22} & ... & z_J\theta_{J2K} \\ ... & ... & ... & ... & ... & ... & ... & ... \\ \theta_{JT_J1} & \theta_{JT_J2} & ... & \theta_{JT_JK} & z_J\theta_{JT_J1} & z_J\theta_{JT_J2} & ... & z_J\theta_{JT_JK} \end{pmatrix}_{N\times 2K} \tag{A.2}$$

$$\boldsymbol{A} = \begin{pmatrix} \mathbf{a}_{11} & 0 & 0 & 0 & ... & \mathbf{a}_{1K} & 0 & 0 & 0 \\ 0 & \mathbf{a}_{21} & 0 & 0 & ... & 0 & \mathbf{a}_{2K} & 0 & 0 \\ 0 & 0 & \ddots & 0 & ... & 0 & 0 & \ddots & 0 \\ 0 & 0 & 0 & \mathbf{a}_{J1} & ... & 0 & 0 & 0 & \mathbf{a}_{JK} \end{pmatrix}_{N\times Q} \tag{A.3}$$

$$\mathbf{a}_{jk} = \begin{pmatrix} \theta_{j1k} \\ \theta_{j2k} \\ \vdots \\ \theta_{jT_jk} \end{pmatrix}$$

For the model with age effect and differential slope, the matrix $\boldsymbol{X}$ and parameter vector $\boldsymbol{\beta}$ should be augmented accordingly without changing matrices $\boldsymbol{A}, \boldsymbol{U}$.

Then $\boldsymbol{Y} \sim N(\boldsymbol{XB}, \boldsymbol{A\Sigma}_u\boldsymbol{A'} + \sigma_0^2\boldsymbol{I_N})$, where

$$\boldsymbol{\Sigma}_u = COV(U) = \begin{pmatrix} \sigma_1^2\boldsymbol{I_J} & 0 & \cdots & 0 \\ 0 & \sigma_2^2\boldsymbol{I_J} & \cdots & 0 \\ \vdots & \vdots & \vdots & \vdots \\ 0 & 0 & \cdots & \sigma_K^2\boldsymbol{I_J} \end{pmatrix}_{Q\times Q} \tag{A.4}$$

Denote $\boldsymbol{V} = \boldsymbol{A\Sigma}_u\boldsymbol{A'} + \sigma_0^2\boldsymbol{I_N}, \boldsymbol{\eta} = (m_1, ..., m_K, \beta_1, ..., \beta_K, \sigma_1^2, ...\sigma_K^2, \sigma_0^2)$.
The complete likelihood function is

$$\begin{aligned} L_0(\boldsymbol{\eta}; \boldsymbol{y}, \boldsymbol{u}) &= f(\boldsymbol{y}|\boldsymbol{u}; \boldsymbol{X}, \boldsymbol{A}, \boldsymbol{\beta}, \boldsymbol{\sigma})f(\boldsymbol{u}; \boldsymbol{X}, \boldsymbol{A}, \boldsymbol{\beta}, \boldsymbol{\sigma}) \\ &= \exp\{-\tfrac{1}{2}[N\ln(2\pi\sigma_0^2) + \tfrac{1}{\sigma_0^2}(\boldsymbol{y} - \boldsymbol{X\beta} - \boldsymbol{Au})'(\boldsymbol{y} - \boldsymbol{X\beta} - \boldsymbol{Au})] \\ &\quad -\tfrac{1}{2}[Q\ln(2\pi) + J\textstyle\sum_{k=1}^K \ln\sigma_k^2 + \boldsymbol{u'\Sigma}_u^{-1}\boldsymbol{u}]\} \end{aligned} \tag{A.5}$$

The complete log likelihood is

$$l(\boldsymbol{\eta}; \boldsymbol{y}, \boldsymbol{u}) \propto -\tfrac{N}{2}log(\sigma_0^2) - \tfrac{1}{2\sigma_0^2}(\boldsymbol{y} - \boldsymbol{X\beta} - \boldsymbol{Au})'(\boldsymbol{y} - \boldsymbol{X\beta} - \boldsymbol{Au}) - \tfrac{J}{2}\textstyle\sum_{k=1}^K \log(\sigma_k^2) - \tfrac{1}{2}\sum_{k=1}^K \tfrac{1}{\sigma_k^2}u_k'u_k \tag{A.6}$$

because

$$\begin{aligned} \boldsymbol{u'\Sigma}_u^{-1}\boldsymbol{u} = (u_1', u_2', ..., u_K') &\begin{pmatrix} \frac{1}{\sigma_1^2}I_J & 0 & 0 & 0 \\ 0 & \frac{1}{\sigma_2^2}I_J & 0 & 0 \\ 0 & 0 & \ddots & 0 \\ 0 & 0 & 0 & \frac{1}{\sigma_K^2}I_J \end{pmatrix} \begin{pmatrix} u_1 \\ u_2 \\ \vdots \\ u_K \end{pmatrix} \\ &= \frac{1}{\sigma_1^2}u_1'u_1 + \frac{1}{\sigma_2^2}u_2'u_2 + ... + \frac{1}{\sigma_K^2}u_K'u_K \\ &= \sum_{k=1}^K \frac{1}{\sigma_k^2}u_k'u_k \end{aligned} \tag{A.7}$$

Let $\boldsymbol{w} = (\boldsymbol{y}, \boldsymbol{u}) := (\boldsymbol{w}_{obs}, \boldsymbol{w}_{mis})$, then

$$\boldsymbol{w}_{obs}|\boldsymbol{w}_{mis} = \boldsymbol{y}|\boldsymbol{u} \sim N(\boldsymbol{X\beta} + \boldsymbol{Au}, \sigma_0^2 \boldsymbol{I}_N)$$

$$\boldsymbol{w}_{mis} = \boldsymbol{u} \sim N_Q(\boldsymbol{0}, \boldsymbol{\Sigma}_u)$$

.

$$\begin{aligned} COV(\boldsymbol{w}_{obs}, \boldsymbol{w}_{mis}) &= COV(\boldsymbol{X\beta} + \boldsymbol{Au} + \boldsymbol{\varepsilon}, \boldsymbol{u}) \\ &= COV(\boldsymbol{Au}, \boldsymbol{u}) \\ &= \boldsymbol{A\Sigma}_u \end{aligned} \tag{A.8}$$

So we have:

$$\begin{pmatrix} \boldsymbol{w}_{obs} \\ \boldsymbol{w}_{mis} \end{pmatrix} = N\left[\begin{pmatrix} \boldsymbol{X\beta} \\ \boldsymbol{0} \end{pmatrix}, \begin{pmatrix} \boldsymbol{V} & \boldsymbol{A\Sigma}_u \\ \boldsymbol{\Sigma}_u \boldsymbol{A}' & \boldsymbol{\Sigma}_u \end{pmatrix}\right]$$

Lemma 2.1. *If* $\boldsymbol{X} = (\boldsymbol{X}_1, \boldsymbol{X}_2)$, *and* $\boldsymbol{X} \sim N[\begin{pmatrix} \boldsymbol{\mu}_1 \\ \boldsymbol{\mu}_2 \end{pmatrix}, \begin{pmatrix} \boldsymbol{\Sigma}_{11} & \boldsymbol{\Sigma}_{12} \\ \boldsymbol{\Sigma}_{21} & \boldsymbol{\Sigma}_{22} \end{pmatrix}]$, *then* $[\boldsymbol{X}_1|\boldsymbol{X}_2] \sim N(\boldsymbol{\mu}_{1|2}, \boldsymbol{\Sigma}_{1|2})$, *where* $\boldsymbol{\mu}_{1|2} = \boldsymbol{\mu}_1 + \boldsymbol{\Sigma}_{12}\boldsymbol{\Sigma}_{22}^{-1}(\boldsymbol{X}_2 - \boldsymbol{\mu}_2)$ *and* $\boldsymbol{\Sigma}_{1|2} = \boldsymbol{\Sigma}_{11} - \boldsymbol{\Sigma}_{12}\boldsymbol{\Sigma}_{22}^{-1}\boldsymbol{\Sigma}_{21}$.

Then we have

$$[\boldsymbol{w}_{mis}|\boldsymbol{w}_{obs}] = [\boldsymbol{u}|\boldsymbol{y}] \sim N_Q(\boldsymbol{\mu}_p, \boldsymbol{\Sigma}_p)$$

and

$$\boldsymbol{\mu}_p = \boldsymbol{\Sigma}_u \boldsymbol{A}'\boldsymbol{V}^{-1}(\boldsymbol{y} - \boldsymbol{X\beta}) \quad \boldsymbol{\Sigma}_p = \boldsymbol{\Sigma}_u - \boldsymbol{\Sigma}_u \boldsymbol{A}'\boldsymbol{V}^{-1}\boldsymbol{A\Sigma}_u$$

Now we let $\boldsymbol{s} = \boldsymbol{Au} + \boldsymbol{X\beta} - \boldsymbol{y}$ and $\boldsymbol{V} := \boldsymbol{A\Sigma}_u\boldsymbol{A}' + \sigma_0^2\boldsymbol{I}_N$. To apply Expectation-Maximization (EM) algorithm, we need to evaluate $E_{\boldsymbol{u}|\boldsymbol{y},\boldsymbol{\eta}}[l(\boldsymbol{\eta}; \boldsymbol{y}, \boldsymbol{u})]$, and consequently $E\big[\boldsymbol{s}'\boldsymbol{s}|\boldsymbol{y}, \boldsymbol{\eta}\big]$, $E\big[\boldsymbol{u}_k'\boldsymbol{u}_k|Y, \boldsymbol{\eta}\big]$.

Then

$$\boldsymbol{s}|(\boldsymbol{y},\boldsymbol{\eta}) \sim N(\boldsymbol{A}\boldsymbol{\mu}_p + \boldsymbol{X}\boldsymbol{B} - \boldsymbol{Y}, \boldsymbol{A}\boldsymbol{\Sigma}_p\boldsymbol{A}')$$

We can estimate parameters iteratively as follows.

E-step:

$$E[\boldsymbol{u}|\boldsymbol{w}_{obs} = \boldsymbol{y}] = \boldsymbol{\Sigma}_u\boldsymbol{A}'\boldsymbol{V}^{-1}(\boldsymbol{y} - \boldsymbol{X}\boldsymbol{\beta})$$

$$E\big[\boldsymbol{s}'\boldsymbol{s}|\boldsymbol{w}_{obs} = \boldsymbol{y}\big] = tr(\boldsymbol{A}\boldsymbol{\Sigma}_p\boldsymbol{A}') + (\boldsymbol{A}\boldsymbol{\mu}_p + \boldsymbol{X}\boldsymbol{\beta} - \boldsymbol{y})'(\boldsymbol{A}\boldsymbol{\mu}_p + \boldsymbol{X}\boldsymbol{\beta} - \boldsymbol{y})$$

$$E\big[\boldsymbol{u}_k'\boldsymbol{u}_k|\boldsymbol{w}_{obs} = \boldsymbol{y}\big] = tr(\boldsymbol{\Sigma}_{p_k}) + \boldsymbol{\mu}_{p_k}'\boldsymbol{\mu}_{p_k}$$

where $\boldsymbol{\mu}_{p_k}$ is the kth diagonal block of matrix $\boldsymbol{\mu}_p$ and $\boldsymbol{\mu}_{p_k}$ is the kth sub-vector in $\boldsymbol{\mu}_p$.

M-step:

$$\hat{\boldsymbol{\beta}}^{(t+1)} = (\boldsymbol{X}'\boldsymbol{X})^{-1}\boldsymbol{X}'(\boldsymbol{y} - \boldsymbol{A}E_{\eta^{(t)}}(\boldsymbol{u}^{(t)}))$$

$$\hat{\sigma}_0^{2(t+1)} = \frac{E_{\eta^{(t)}}\big[\boldsymbol{s}'\boldsymbol{s}|\boldsymbol{w}_{obs} = \boldsymbol{y}\big]}{N}$$

$$\hat{\sigma}_k^{2(t+1)} = \frac{E_{\eta^{(t)}}\big[\boldsymbol{u}_k'\boldsymbol{u}_k|\boldsymbol{w}_{obs} = \boldsymbol{y}\big]}{J}$$
